# Field Strength‐Dependent White Matter *R*_1_ and *R*_2_ Anisotropy of Phase‐Cycled Balanced Steady‐State Free Precession Relaxometry

**DOI:** 10.1002/mrm.70255

**Published:** 2026-01-23

**Authors:** Florian Birk, Hamzeh Tesh, Ali Aghaeifar, Svenja Klinkowski, Praveen Iyyappan Valsala, Sebastian Mueller, Svenja Brodt, Klaus Scheffler, Rahel Heule

**Affiliations:** ^1^ High‐Field Magnetic Resonance Max Planck Institute for Biological Cybernetics Tübingen Germany; ^2^ Department of Biomedical Magnetic Resonance University of Tübingen Tübingen Germany; ^3^ Brain States for Plasticity Max Planck Institute for Biological Cybernetics Tübingen Germany; ^4^ Institute of Medical Psychology and Behavioral Neurobiology University of Tübingen Tübingen Germany; ^5^ Center for MR Research University Children's Hospital Zurich Switzerland

**Keywords:** brain tissue anisotropy, orientation dependence, phase‐cycled bSSFP, relaxometry, spin walk, white matter

## Abstract

**Purpose:**

To investigate how the relaxation rates (*R*_1_, *R*_2_) and asymmetry indices (AI), derived from phase‐cycled balanced steady‐state free precession (pc‐bSSFP) data, depend on the orientation of white matter (WM) fiber tracts at different field strengths.

**Methods:**

Phase‐cycled bSSFP data acquired at 3 and 9.4T in the healthy human brain were processed using motion‐insensitive rapid configuration relaxometry (MIRACLE) and a frequency response analysis to derive *R*_1_, *R*_2_, and AI values, respectively. Fractional anisotropy (FA) and fiber‐to‐field angle (*θ*) were estimated based on 3T diffusion tensor imaging. The orientation dependence of *R*_1_, *R*_2_, and AI in WM was characterized using literature model fits as well as Monte Carlo random walk simulations to explore the influence of field strength and susceptibility effects.

**Results:**

*R*_2_ and AI exhibited a pronounced orientation dependence while the influence of anisotropy on *R*_1_ was weaker, but noticeable. The observed anisotropy increased systematically from 3 to 9.4T. Literature models assuming either a susceptibility or a generalized magic angle effect described the *R*_2_ and AI anisotropy to a high degree (*R* ^2^ ≥ 0.99). The calculated partial contributions of susceptibility to *R*_2_ anisotropy increased from 24.0%–39.0% at 3T to 77.0%–87.1% at 9.4T. The Monte Carlo simulations were able to reproduce the characteristics of *R*_2_ anisotropy, but not its strength.

**Conclusion:**

Microstructure‐driven relaxation anisotropy considerably affects pc‐bSSFP relaxometry, in particular *R*_2_. The findings indicate that *R*_2_ anisotropy is driven by susceptibility at ultra‐high fields whereas additional mechanisms likely contribute at lower field strengths.

## 1 Introduction

The tightly packed bundles of myelinated axons in white matter (WM) create structural anisotropy that restricts water diffusion, with myelin water confined between phospholipid bilayers exhibiting highly restricted mobility [1]. This confined microstructural organization generates differences between myelin water and intra‐axonal or extra‐axonal water that are detectable with quantitative magnetic resonance imaging (qMRI) techniques, providing a non‐invasive way to assess microstructural changes in neurodegenerative diseases such as multiple sclerosis (MS) [2, 3, 4, 5, 6, 7].

The balanced steady‐state free precession (bSSFP) signal, which is known to be highly frequency‐sensitive, was reported to reflect tissue microarchitecture and biochemical composition on a subvoxel level [8, 9, 10, 11]. While homogeneous media characterized by a single longitudinal ($R_{1}=1/T_{1}$) and transverse ($R_{2}=1/T_{2}$) relaxation component and a single resonance frequency produce symmetric bSSFP frequency responses, tissues with microstructural boundaries and multiple compartments (e.g., in the presence of myelin, iron, deoxyhemoglobin, or lipids) create asymmetric profiles [8, 12]. Particularly pronounced asymmetries were found in WM, demonstrating a distinct dependence on fiber orientation, with largest asymmetries occurring in highly anisotropic tracts oriented perpendicular to the main magnetic field (*B*_0_) [9, 10, 13, 14]. This points to diamagnetic myelin, which is susceptibility‐shifted with respect to the water molecules in the intra‐ and extra‐axonal spaces, in combination with multi‐component relaxation as possible physical mechanisms underlying the observed asymmetries [12, 15, 16].

Since accurate modeling of the intra‐voxel frequency distribution is challenging, current phase‐cycled bSSFP (pc‐bSSFP) relaxometry methods employ simplified single‐component models that underestimate relaxation times compared to standard spin‐echo or inversion‐recovery techniques [15, 16, 17, 18, 19]. Nonetheless, the derived parameters carry inherent information about myelin content and fiber directionality, which may serve as a marker of tissue integrity. In addition, pc‐bSSFP enables simultaneous quantification of *R*_1_ and *R*_2_ relaxation rates with high signal‐to‐noise ratio, 3D sampling efficiency, and robustness to frequency shifts induced by macroscopic *B*_0_ field variations, thus presenting a popular choice for multi‐parametric relaxometry [15, 16, 17, 18, 19, 20, 21].

Relaxation anisotropy with a dependence on fiber‐to‐field angle (*θ*) is a well‐known phenomenon, particularly for *R*_2_* (1/*T*_2_*) mapping in WM, which exhibits a strong orientation dependence, predominantly attributed to the structural anisotropy of the axons in conjunction with susceptibility anisotropy of the lipid molecules within the myelin sheath [22, 23, 24, 25, 26, 27, 28, 29, 30, 31, 32, 33, 34, 35]. Furthermore, it is well established that dipole–dipole interactions in highly ordered collagen‐rich fibers such as tendons [36, 37], cartilage [38, 39], and peripheral nerves [40] produce *R*_2_ (and consequently *R*_2_*) anisotropy with a minimum at the magic angle (θ=54.7°). More recently, anisotropy of *R*_2_ relaxation was observed in WM in both adult and newborn brains with likely multiple underlying biophysical mechanisms related to diffusion‐mediated dephasing in susceptibility‐induced inhomogeneous fields or residual dipolar coupling between motion restricted protons [41, 42, 43, 44, 45].

Contrary to the tendency to characterize WM *R*_2_ and *R*_2_* anisotropy predominantly through susceptibility‐based mechanisms, recent studies have proposed a generalized magic angle effect (MAE) model to explain the underlying mechanism [46, 47, 48, 49]. Unlike cartilage, where dipolar interactions follow a (3cos^2^ *θ* − 1)^2^ dependence on fiber direction, WM involves residual dipolar couplings (RDCs) perpendicular or parallel to the myelin bilayer surface normal, arising from ordered water molecules located between the phospholipid bilayers and semisolid methylene protons within the bilayers [47, 48]. Apart from the myelin sheath, ordered water may also exist in the intra‐axonal compartment. This requires extended MAE models incorporating the angle α between couplings and the axon axis as well as the offset angle ε_0_ between principal and apparent (axial + radial) diffusion directions to account for non‐zero diffusion perpendicular to the axons [47, 48, 49].

For *R*_1_, a relatively weak angular dependence in WM was discovered recently and exhibited characteristics of a magic angle rather than susceptibility effect [50, 51, 52, 53, 54, 55]. Combined contrasts like *R*_1_/*T*_2_*, leveraging both susceptibility and magic angle mechanisms, were reported to enhance WM anisotropy [56].

Here, we evaluate the orientation dependence of *R*_1_, *R*_2_, and the asymmetry index (AI) derived from pc‐bSSFP in healthy human brain WM at 3 and 9.4T. We analyze the field strength dependence and potential mechanisms contributing to the observed anisotropy, such as susceptibility or MAE, by fitting proposed literature models to the in vivo data and performing Monte Carlo simulations for realistic WM axon models (AM) using SpinWalk [57].

## 2 Methods

### 2.1 MR Data Acquisition

MR data were acquired in‐house within a previous small‐cohort 3T (3T_SC_)/9.4T (9.4T_SC_) study and a large‐cohort 3T (3T_LC_), with ethical approval and written informed consent obtained from all participants prior to MRI. The small‐cohort data included six healthy adults (3 male, 3 female, age: 30.3 ± 6.5 years) measured both at 3T (Magnetom Prisma) and at 9.4T (Magnetom 9.4T, Siemens Healthineers, Erlangen, Germany/custom 18‐transceiver/14‐receive‐only head array coil [58]) published previously [14]. The utilized large‐cohort data included the baseline measurements of 122 healthy adults (57 male, 65 female, age: 23.3 ± 3.5 years) scanned at 3T (Magnetom Prisma, Siemens Healthineers, Erlangen, Germany/vendor‐provided 64‐channel receive head array coil) within a recently published multi‐contrast MRI study [59]. The primary focus was to investigate pc‐bSSFP relaxation anisotropy and its field strength dependence to reveal the underlying biophysical mechanisms. The 3T large cohort (3T_LC_) was evaluated additionally since it presented a unique dataset with expected increased robustness and reliability for model fitting at 3T, whereas it is challenging to establish a comparably large cohort at ultra‐high‐field strength.

The protocols included customized whole‐brain 3D sagittal pc‐bSSFP acquisitions for joint *R*_1_ and *R*_2_ relaxometry with nominal isotropic resolutions of 1.4 × 1.4 × 1.4 mm^3^ (3T_LC_), 1.3 × 1.3 × 1.3 mm^3^ (3T_SC_), and 0.8 × 0.8 × 0.8 mm^3^ (9.4T_SC_). Phase‐cycled bSSFP employed a 12‐point phase‐cycling scheme with radiofrequency (RF) phase increments *ϕ*_j_ = *π*/12·(2*j* − 1), *j* = 1,2, … 12, uniformly distributed over (0, 2*π*) at both field strengths, and 256 dummy pulses preceding each RF cycle to ensure steady‐state conditions. Transmit field (B_1_^+^) inhomogeneity correction was based on a rapid 2D multi‐slice TurboFLASH sequence (3T) [60, 61] or actual flip angle imaging (AFI) (9.4T) [62]. Diffusion tensor imaging (DTI) data were acquired either using a multi‐band‐accelerated 2D multi‐slice single‐shot spin‐echo echo‐planar‐imaging (SE‐EPI) in oblique (3T_LC_) [63] or 2D multi‐slice single‐shot SE‐EPI in strictly axial (3T_SC_) orientation. The MR protocols were complemented by vendor‐provided anatomical 3D T_1_‐weighted MP2RAGE [64] (1.0 × 1.0 × 1.0 mm^3^, 3T_LC_) or MPRAGE [65] scans (1.2 × 1.2 × 1.2 mm^3^, 3T_SC_). The relevant protocol parameters of those sequences are provided in detail in Table 1.

**TABLE 1:** Relevant sequence parameters of the MR protocols for the large‐cohort (LC, *n* = 107) study at 3T (3T_LC_) and the small‐cohort (SC, *n* = 6) study at 3T (3T_SC_)/9.4T (9.4T_SC_).

Sequence	Dataset	Resolution (field of view) [mm × mm × mm]	TR/TE [ms]	Nominal flip angle *α*_nom_ [°]	GRAPPA acceleration (in‐plane/through‐plane)	Scan time [min]^a^
pc‐bSSFP	3T_LC_	1.4 × 1.4 × 1.4 (224 × 224 × 179)	4.8/2.4	15	2 (2/1)	9:25
	3T_SC_	1.3 × 1.3 × 1.3 (229 × 229 × 166)	4.8/2.4	15	2 (2/1)	10:12
	9.4T_SC_	0.8 × 0.8 × 0.8 (220 × 220 × 166)	4.0/2.0	9	4 (2/2)	9:17
TFL B_1_^+^	3T_LC_	2.4 × 2.4 × 3.0 (229 × 229 × 90)	14180/2.37	8	—	0:29
	3T_SC_	2.4 × 2.4 × 3.0 (229 × 229 × 90)	14180/2.37	8	—	0:29
AFI B_1_^+^	9.4T_SC_	2.3 × 2.3 × 3.0 (220 × 220 × 168)	20, 100/4	60	4 (2/2)	2:07

Sequence	Dataset	Resolution (field of view) [mm × mm × mm]	TR/TE [ms]	Nominal flip angle *α*_nom_ [°]	Inversion time [ms]	Scan time [min]
MP2RAGE	3T_LC_	1.0 × 1.0 × 1.0 (256 × 256 × 176)	5000/2.98	4/5	700/2500	8:52
MPRAGE	3T_SC_	1.2 × 1.2 × 1.2 (144 × 229 × 186)	2200/3.44	8	900	3:17

Sequence	Dataset	Resolution (field of view) [mm × mm × mm]	TR/TE [ms] acceleration mode (acceleration factor)	Diffusion gradient scheme/number of diffusion directions	*b*‐values [s/mm^2^]	Scan time [min] (phase encoding direction)
SE‐EPI DTI	3T_LC_	2.0 × 2.0 × 2.0 (140 × 220 × 220)	3000/53 GRAPPA (2), Multi‐band (2)	Monopolar/35	0, 1000	2:18 (AP), 2:18 (PA)
SE‐EPI DTI	3T_SC_	1.4 × 1.4 × 3.0 (108 × 229 × 184)	4800/83 GRAPPA (2), partial Fourier 6/8	Bipolar/20	0, 1000	15:23 (RL), 0:59 (LR)

*Note*: Phase‐cycled balanced steady‐state free precession (pc‐bSSFP) parameters were identical for the 3T_LC_ and 3T_SC_ protocols except for spatial resolution. B_1_^+^ correction employed a 2D multi‐slice TurboFLASH sequence at 3T and actual flip angle imaging (AFI) at 9.4T. Anatomical reference images were acquired using MP2RAGE (3T_LC_) and MPRAGE (3T_SC_) sequences. Diffusion tensor imaging (DTI) was based on 2D multi‐slice single‐shot spin‐echo echo‐planar‐imaging (SE‐EPI) for both 3T protocols with additional multi‐band acceleration in case of the 3T_LC_ study.

^a^ For the pc‐bSSFP acquisition, the number of slices per slab and thus the scan time was slightly increased in a few cases to ensure whole‐brain coverage.

### 2.2 MR Data Processing

All data underwent quality control, resulting in the inclusion of 107/122 (exclusions due to missing data, motion artifacts, or suspected incidental findings) subjects from the 3T_LC_ study and 5/6 subjects from the 3T_SC_/9.4T_SC_ study (exclusion due to missing B_1_^+^ map at 3T). The effect of field strength was analyzed based on the five subjects with complete data at both 3 and 9.4T.

For all registration and resampling steps, SimpleITK (v2.0.0) [66] was used. Structural T_1_‐weighted datasets were skull‐stripped (SynthStrip v1.0 [67]), segmented (SynthSeg v2.0 [68]), and subsequently resampled to match the pc‐bSSFP resolution. Each complex‐valued 3D bSSFP volume was co‐registered to the middle 165° phase‐cycle used as reference, by calculating the transformations for the magnitude data and then applying them to the real and imaginary data. Coil phase averaged across RF phase increments was subtracted voxel‐wise to eliminate receiver‐related offsets, followed by ringing artifact removal using local subvoxel shifts [69]. A discrete Fourier transform was applied to the intra‐registered complex‐valued pc‐bSSFP data to extract the three lowest‐order SSFP configurations (F_−1_, F_0_, and F_1_) as Fourier coefficients [70], each exhibiting distinct T_1_ and T_2_ sensitivity. Base B_1_^+^ acquisition data was skull‐stripped and registered to the mean of the intra‐registered pc‐bSSFP magnitude data, with subsequent application of the obtained transformations to the quantitative actual flip angle maps. Post‐registration B_1_^+^ maps were 3D median‐filtered (kernel: 15 × 15 × 15 voxels) to remove any residual structural bias. The mean pc‐bSSFP magnitude was then registered to the anatomical T_1_‐weighted reference and the transformation was applied to the complex‐valued (real/imaginary) pc‐bSSFP data, complex‐valued (real/imaginary) SSFP configurations, and B_1_^+^ maps. In the case of the 9.4T_SC_ data, the 3T_SC_ T_1_‐weighted dataset was used as reference with otherwise identical processing as described above.

The relaxation rates (*R*_1_, *R*_2_) were derived from the pc‐bSSFP data using motion‐insensitive rapid configuration relaxometry (MIRACLE) with B_1_^+^ correction [15], by taking the *F*_−1_, *F*_0_, and *F*_1_ magnitudes as input. The AI was calculated from the normalized *B*_0_‐corrected bSSFP frequency response as $\mathrm{AI}=\left(h_{p}-h_{n}\right)/\left(h_{p}+h_{n}\right)$, where *h*_p_ and *h*_n_ represent the signal peaks at positive and negative frequency offsets [9].

DTI base data were processed using FSL, including the correction of susceptibility distortions (TOPUP [71]) and eddy current distortions (EDDY [72]), followed by diffusion tensor fitting (DTIFIT). Fractional anisotropy (FA) and fiber‐to‐field angle (*θ*) maps were calculated from the Eigenvalues and principal Eigenvector of the diffusion tensor, respectively. In case of oblique scans, the principal Eigenvector was rotated into the scanner coordinate system. Orientation dependence of *R*_1_, *R*_2_, and AI in WM was systematically evaluated by binning WM voxels into different FA and *θ* intervals. For this purpose, whole‐brain WM masks were generated from SynthSeg [68] cerebral WM labels and refined using histogram‐based intensity thresholds to exclude outliers (see Figure S1 and Supporting Information: Section White matter mask). WM voxels were analyzed using three binning strategies: (1) FA bins spanning 0.0–1.0 (0.2 step size) and θ bins spanning 0°–90° (7.5° step size); (2) high (0.5–1.0) FA thresholding with fine *θ* sampling over 0°–90° (2.5° step size); and (3) fine resolution with FA bins spanning 0.0–0.8 (0.05 step size) and *θ* bins spanning 15°–90° (5° step size).

### 2.3 Relaxation Anisotropy Models

To explore mechanisms underlying qMRI anisotropy in WM, mean inter‐subject *R*_1_, *R*_2_, and AI values (binning strategy (2), high FA interval) were fitted to established literature models:

(1) $$\text{Susceptibility model}:y\left(\theta \right)=a+b\cdot \sin^{2}\theta +c\cdot \sin^{4}\theta$$

(2) $$\text{Classical dipole}:y\left(\theta \right)=a+b\cdot \left(3\cos^{2}\theta -1\right)$$

(3) $$\text{Extended dipole}:y\left(\theta \right)=a+b\cdot {\left(3\cos^{2}\theta -1\right)}^{2}$$

and

(4) $$\text{Generalized magic angle effect}:y\left(\theta \right)=p_{i}+p_{a}\cdot f\left(\alpha ,\theta -\varepsilon_{0}\right)$$

with

(5) $$\begin{array}{cc} f\left(\alpha ,\varepsilon \right) & =\frac{1}{4}\cdot {\left(3\cos^{2}\alpha -1\right)}^{2}\cdot {\left(3\cos^{2}\varepsilon -1\right)}^{2}+\frac{9}{8}\\ & \;\cdot \left(\mathrm{si}n^{4}\alpha \cdot \mathrm{si}n^{4}\varepsilon +\mathrm{si}n^{2}2\alpha \cdot \mathrm{si}n^{2}2\varepsilon \right) \end{array}$$

Equation (1) represents the magnetic susceptibility model with *θ*‐dependent parameters, either attributed to the interaction between susceptibility differences and applied field gradients (sin^2^ *θ*) as well as diffusion‐mediated decoherence in inhomogeneous fields (sin^4^ *θ*) suggested for spin‐echo *R*_2_ anisotropy [42] or attributed to *R*_2_* orientation dependence based on susceptibility theory under the assumption of an anisotropic myelin susceptibility accounting for the higher‐order term (sin^4^ *θ*) [73, 74]. Equations (2) and (3) describe classical dipole–dipole interactions like the MAE [75, 76] and extended dipole–dipole interaction models [40, 73, 77], primarily used to describe orientation‐dependent MR signals or *R*_2_ rates for peripheral nerves [40] and collagen [77]. While *R*_1_ has not been fitted to any of those models, a MAE has been proposed as source in several previous studies [50, 51, 52, 53, 54, 55]. A co‐existence of susceptibility and magic angle effects results in a general model equivalent to Equation (1) [73]. Equations (4) and (5) represent a generalized MAE model for anisotropic transverse relaxation in WM, where α describes the angle between RDCs of ordered water molecules and the axon fiber, and *ε* ($=\theta -\varepsilon_{0}$) accounts for the angle between the actual (rather than principal) fiber direction and *B*_0_ [47, 48, 49]. Parameters *p*_i_ and *p*_a_ represent isotropic (*θ*‐independent) and anisotropic (*θ*‐dependent) components (*p* = *R*_1_, *R*_2_, AI, depending on the fitted data). While the three models in Equations ((1), (2), (3)) were fitted without constraints, the generalized MAE model (Equations 4 and 5) employed biologically informed bounds for parameter identifiability and numerical stability [47, 49]: *p*_i_ = [0, 100] 1/s, *p*_a_ = [0, 50] 1/s, *α* = [0°, 90°], and *ε*_0_ = [0°, 45°] in case of *R*_1_ and *R*_2_ fits. For AI fits, identical α and ε_0_ constraints were used, but the isotropic (*p*_i_) and anisotropic (*p*_a_) components were unconstrained to allow negative values. Additionally, the generalized MAE model (Equations 4 and 5) was fitted by estimating *ε*_0_ directly voxel‐wise from the in vivo DTI data as arctan(RD/AD) (RD: radial diffusivity, AD: axial diffusivity) [47, 48]. The estimated *ε*_0_ was then subtracted from θ voxel‐wise and the data divided into *ε* ($=\theta -\varepsilon_{0}$) bins instead of *θ* bins, thus eliminating *ε*_0_ as free fitting parameter in the model (referred to as generalized MAE model with *ε*_0_ correction). In case of Monte Carlo spin simulations (see section below), *ε*_0_ was set to zero. Data fitting employed the *curve_fit* method from *scipy.optimize* [78], with goodness of fit evaluated using the coefficient of determination $R^{2}=1-\frac{\sum \left(y-\hat{y}\right)2}{\sum \left(y-\bar{y}\right)2}$, where $\hat{y}$ represents model predictions and $\bar{y}$ the data mean.

### 2.4 Quantification of Anisotropy and Susceptibility Contribution

The anisotropy, that is, dependence on fiber‐to‐field angle, was quantified as percentage change for each subject and then averaged across all subjects. *R*_1_ and *R*_2_ anisotropy was calculated as: $\left(R_{i,\max}-R_{i,\min}\right)/\left(R_{i,\max}+R_{i,\min}\right)\times 100$ (with *i* = 1, 2 for *R*_1_, *R*_2_), where *R*_*i*,max_ and *R*_*i*,min_ represent maximum and minimum values across fiber‐to‐field angle bins (0°–90°, 2.5° step size). For the inherently normalized AI, anisotropy was calculated as: $\left({\mathrm{AI}}_{\max}-{\mathrm{AI}}_{\min}\right)\times 100$. Similarly, anisotropy of the field strength difference ($\Delta =9.4T_{\mathrm{SC}}-3T_{\mathrm{SC}}$) was derived as $\left({\Delta R}_{i,\max}-{\Delta R}_{i,\min}\right)/\left({\Delta R}_{i,\max}+{\Delta R}_{i,\min}\right)\times 100$ for *R*_1_ and *R*_2_ (*i* = 1, 2) and as $\left({\mathrm{\Delta AI}}_{\max}-{\mathrm{\Delta AI}}_{\min}\right)\times 100$ for AI.

The partial contribution of susceptibility to the anisotropic *R*_2_ component $\left(R_{2,a}\right)$ was quantified as $100\cdot {\Delta R}_{2,a}/\left(R_{2,a}\left(3\;T\right)\times \left(\eta -1\right)\right)$ for 3T and $100\cdot \left(\eta \times {\Delta R}_{2,a}\right)/\left(R_{2,a}\left(9.4T\right)\times \left(\eta -1\right)\right)$ for 9.4T, with ${\Delta R}_{2,a}=R_{2,a}\left(9.4\;T\right)-R_{2,a}\left(3\;T\right)$ and $\eta ={\left(9.39/2.89\right)}^{2}=10.56$ (exact nominal squared field strength ratio corresponding to in vivo and in silico data), according to Refs [49, 79]., with $R_{2,a}$ obtained from fitting Equation (4).

### 2.5 Monte Carlo Spin Simulations

Monte Carlo simulations of randomly diffusing spins were performed using the SpinWalk toolbox [57]. The three‐compartment hollow‐cylinder axon model [28] from the AxonPacking toolbox [80] represented nerve fibers as infinite cylinders with myelin (outer cylinder), intra‐axonal (inner cylinder), and extra‐axonal water compartments. Axon radii were gamma‐distributed (mean = 0.7 μm, variance = 0.25 μm, range 0.25–5 μm) [81, 82, 83] with g‐ratios uniformly sampled between 0.6 and 0.7 [84, 85, 86]. A total of 1000 axons were packed as 2D fiber disks into 43 × 43 μm^2^ with adjustable dropout levels of axons after packing (AM_100_ = 0%, AM_75_ = 25%, AM_50_ = 50% dropout), using AM_75_ as default. Analytical field perturbations were computed for fiber orientations from *θ* = 0° to 90° (5° increments), using a default susceptibility shift of Δ*χ* = −0.1 ppm for myelin relative to intra−/extra‐axonal water, applied to both isotropic and anisotropic susceptibility components [12, 28, 87] (see Supporting Information: Section Monte Carlo spin simulations). Subsequently, 2D slices of axon disks were extruded into 3D masks and magnetic field maps for SpinWalk input. Compartments were treated as impermeable, enabling compartmental analysis of orientation dependence. Monte Carlo spin‐walks employed 10^5^ spins uniformly distributed across the AM, repeated for 19 fiber‐to‐field angles covering 0°–90° at 12 phase cycles each to sample the bSSFP frequency response (same phase‐cycling scheme as used in vivo). Default 3 and 9.4T SpinWalk configurations were set up with AM_75_, realistic relaxation rates, diffusivities, and susceptibility shift, as well as pc‐bSSFP parameters (TR/TE, flip angle) matched to the respective in vivo protocols (cf. Figure S1 and Table 2). In total, 11 different SpinWalk configurations were run with identical spin starting positions, by varying *B*_0_, TR/TE, Δ*χ*, diffusivity, and axon models (cf. Table 2). To isolate the effect of *B*_0_, one 9.4T configuration was run with relaxation rates and pc‐bSSFP parameters matched to the default 3T configuration (cf. configuration 9.4T *B*_0_‐only in Table 2). Given myelin volume fractions of 39.2% (AM_100_), 30.3% (AM_75_), and 21.1% (AM_50_), effective myelin proton density was set to one‐half [84, 95, 96] of the extra−/intra‐axonal proton density, yielding realistic myelin water fractions of 24.5%, 17.9%, and 11.9%, respectively [3, 28, 45]. The obtained complex‐valued pc‐bSSFP simulation data were then subjected to MIRACLE for *R*_1_ and *R*_2_ quantification.

**TABLE 2:** Different SpinWalk configurations for anisotropy analysis of in silico pc‐bSSFP relaxometry, based on a three‐compartment WM axon model (AM) with extra‐axonal (EA), intra‐axonal (IA), and myelin compartments.

Configuration	*B*_0_ [T]^a^	TR/TE [ms]	Flip angle *α* [°]	Δ*χ* [ppm]	Diffusivity EA/ IA/myelin [m^2^/s]	Axon model
3T default	**2.89**	**4.8/2.4**	**15**	**−0.1**	**1e−9/1e−9/1e−10**	**AM**_**75**_
9.4T default	**9.39**	**4.0/2.0**	**9**	**−0.1**	**1e−9/1e−9/1e−10**	**AM**_**75**_
3T Diff: 0	2.89	4.8/2.4	15	−0.1	**0.0/0.0/0.0**	AM_75_
9.4T Diff: 0	9.39	4.0/2.0	9	−0.1	**0.0/0.0/0.0**	AM_75_
3T AM_100_	2.89	4.8/2.4	15	−0.1	1e−9/1e−9/1e−10	**AM**_**100**_
3T AM_50_	2.89	4.8/2.4	15	−0.1	1e−9/1e−9/1e−10	**AM**_**50**_
3T Δ*χ*:−2e−7	2.89	4.8/2.4	15	**−0.2**	1e−9/1e−9/1e−10	AM_75_
9.4T Δ*χ*:−2e−7	9.39	4.0/2.0	9	**−0.2**	1e−9/1e−9/1e−10	AM_75_
3T TR10	2.89	**10.0/5.0**	15	−0.1	1e−9/1e−9/1e−10	AM_75_
3T Diff_myelin_: 0	2.89	4.8/2.4	15	−0.1	**1e−9/1e−9/0.0**	AM_75_
9.4T *B*_0_‐only	**9.39**	4.8/2.4	15	−0.1	1e**−**9/1e**−**9/1e**−**10	AM_75_

*Note*: Default 3 and 9.4T configurations replicate the in vivo pc‐bSSFP protocols with realistic tissue relaxation times at 3T of *T*_1_/*T*_2_ = 1000/60, 1000/60, and 300/12 ms, and at 9.4T of *T*_1_/*T*_2_ = 1500/30, 1500/30, and 400/10 ms for EA, IA, and myelin compartments [45, 84, 88, 89, 90, 91], respectively, and diffusivities of *D*_EA/IA_ = 1 × 10^−9^ m^2^/s, *D*_myelin_ = 0.1 × 10^−9^ m^2^/s [92, 93, 94]. Systematic variations include setting the diffusivity to zero (all compartments (Diff: 0) or myelin‐only (Diff_myelin_: 0)), different axon models (AM_100_: 0% dropout, AM_50_: 50% dropout), altered magnetic susceptibility difference (Δ*χ* = −2 × 10^−7^), prolonged repetition and echo times (TR/TE: 10/5 ms), and a hybrid configuration with 9.4T field strength, but 3T sequence parameters and relaxation rates to isolate field‐dependent effects (*B*_0_‐only). The 3 and 9.4T default configurations as well as all changes with respect to the default configurations are highlighted in bold.

^a^ Nominal field strengths corresponding to the in vivo 3/9.4T data.

## 3 Results

**FIGURE 1:**
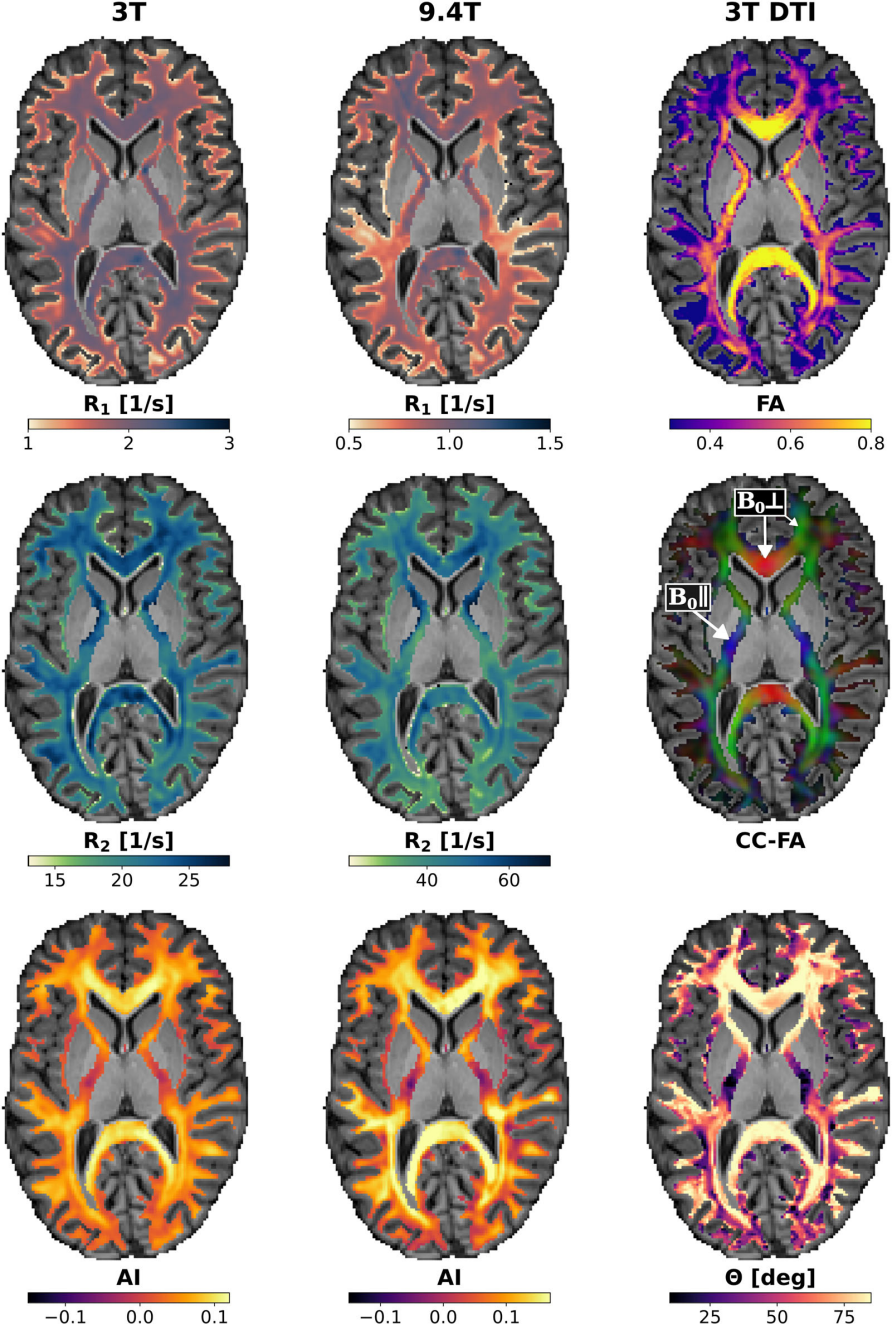
Representative axial slices of quantitative parameter maps within white matter masks overlaid onto anatomical *T*_1_‐weighted data from a subject of the small cohort measured at both field strengths. Longitudinal relaxation rate (*R*_1_), transverse relaxation rate (*R*_2_), and asymmetry index (AI) maps are shown for 3T (left column) and 9.4T (middle column). Recommended colormaps are used for *R*_1_ and *R*_2_ [97]. Diffusion tensor imaging (DTI)‐derived metrics acquired at 3T (right column) include fractional anisotropy (FA), color‐coded FA (CC‐FA), and fiber‐to‐field angle (*θ*). The CC‐FA represents the color‐coded principal diffusion direction map derived from the first eigenvector (V1) of the diffusion tensor and weighted by FA, reflecting fiber directionality along the scanner coordinate system (red: Right–left, green: Anterior–posterior, blue: Superior–inferior/*B*_0_ direction). White arrows in the CC‐FA map indicate representative fiber tracts oriented parallel and perpendicular to *B*_0_. The fiber‐to‐field angle *θ* represents the angle between V1 and *B*_0_, assuming white matter voxels contain a single predominant fiber population with coherent directionality.

Representative relaxation rate (*R*_1_, *R*_2_) and AI maps obtained at 3T (left column) and 9.4T (middle column) are presented in Figure 1 for WM masks overlaid onto anatomical T_1_‐weighted data in an axial slice from one subject measured at both field strengths. Corresponding DTI metrics acquired at 3T, including FA, color‐coded FA (CC‐FA), and fiber‐to‐field angle *θ* (right column), serve as reference for assessing orientation and fractional anisotropy of different WM fiber tracts. Visual examination of Figure 1 reveals two key findings: first, *R*_2_ and AI maps exhibit a pronounced dependence on fiber orientation relative to *B*_0_ in highly anisotropic (i.e., high FA) WM structures (cf. white arrows in CC‐FA pointing to parallel vs. perpendicular tracts with respect to *B*_0_); second, this angular dependence is enhanced at 9.4T relative to 3T.

**FIGURE 2:**
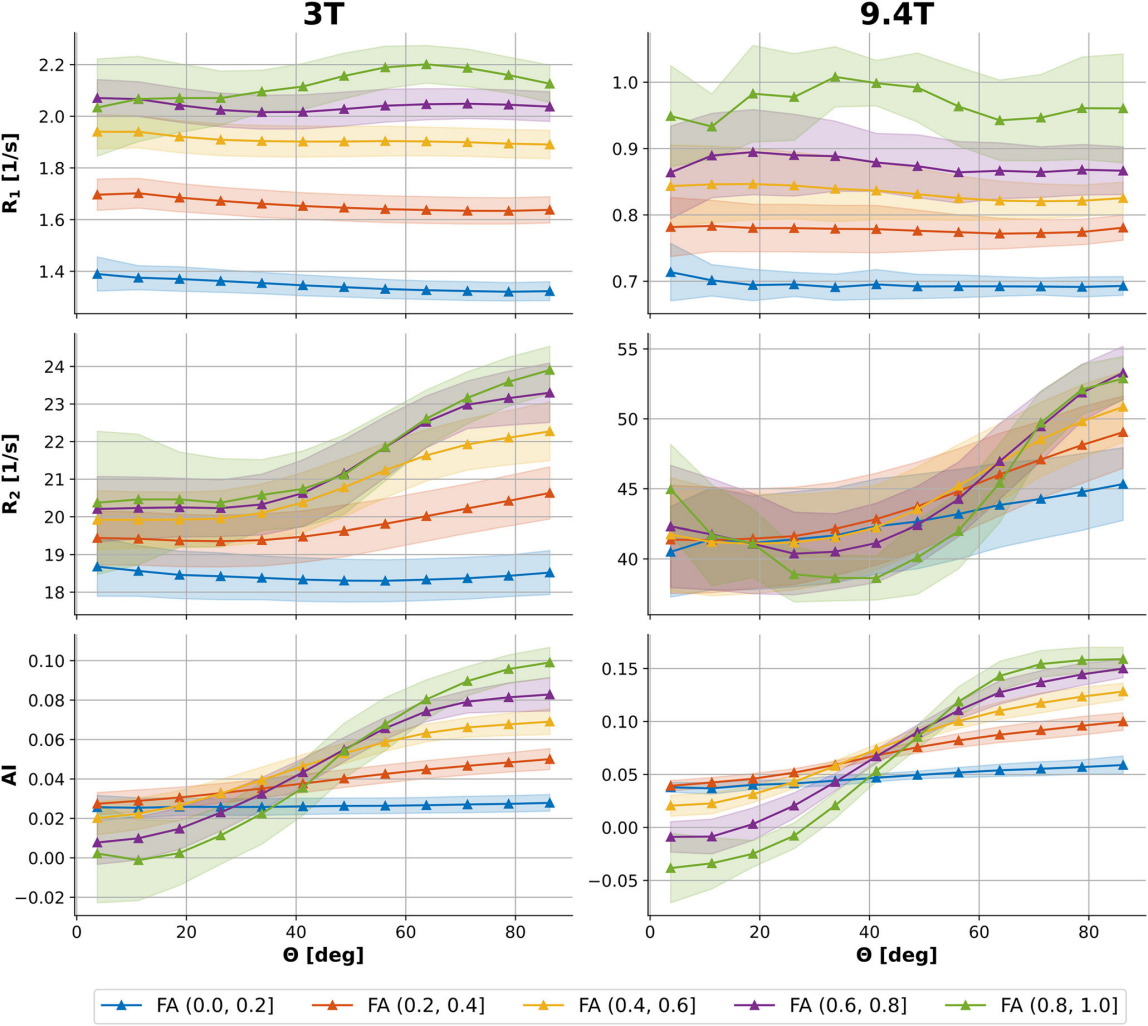
2D visualization of *R*_1_ (first row), *R*_2_ (second row), and AI (third row) anisotropy in global white matter versus fiber‐to‐field angle (*θ*) for different fractional anisotropy (FA) ranges. Using binning strategy (1), WM voxels were binned into five FA intervals, that is (0.0, 0.2], (0.2, 0.4], (0.4, 0.6], (0.6, 0.8], and (0.8, 1.0], using *θ* bins spanning 0°–90° with a step size of 7.5°. Inter‐subject mean values ± standard deviations obtained from the large 3T cohort (left column) and small 9.4T cohort (right column) are displayed for each bin. Triangle markers indicate mean values at the center of each *θ* bin, with shaded areas representing inter‐subject standard deviation.

Binning analysis of global WM versus *θ* and FA in Figure 2 (2D illustration, binning strategy (1)) and Figure S2 (3D illustration, binning strategy (3)) shows that *R*_1_ scales considerably with tissue anisotropy (FA), while its variation with *θ* is minimal and becomes appreciable only in highly anisotropic regions (FA > 0.8; green curves in Figure 2). An increase of both *R*_1_ and *R*_2_ with higher FA can be observed in WM tracts perpendicular to *B*_0_ (for θ=90°). Additionally, *R*_2_ and AI exhibit a clear dependence on fiber orientation, which is enhanced for bins with higher FA values where WM voxels are dominated by a single fiber direction. Furthermore, it can be observed that the *R*_2_ relaxation anisotropy changes its characteristics at 9.4T compared to 3T, exhibiting a quadratic *θ*‐dependence, in particular for high FA.

**FIGURE 3:**
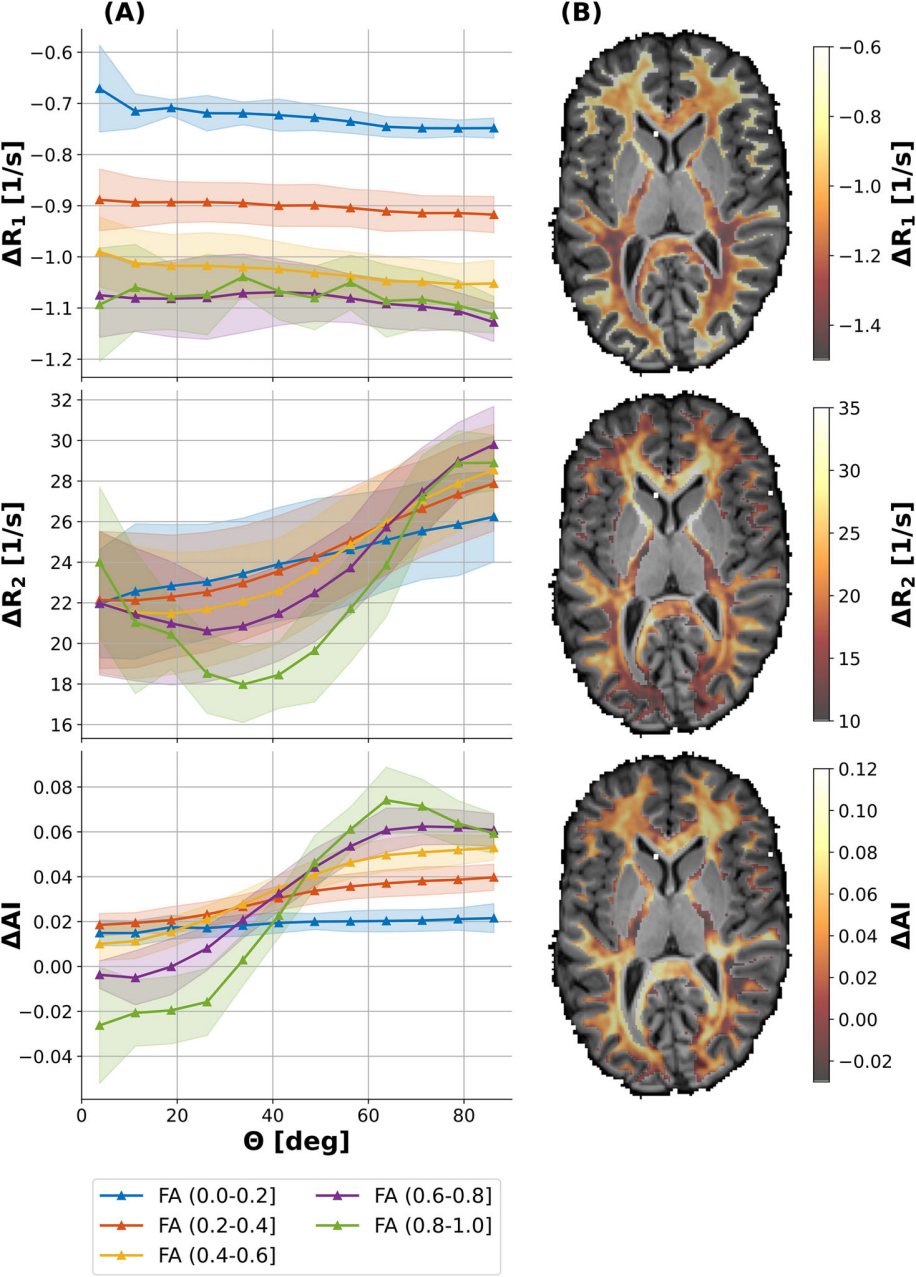
Field strength difference analysis to assess field strength‐dependent *R*_1_, *R*_2_, and AI anisotropy. (A) Mean field strength differences of the subject‐matched small cohort (Δ = 9.4T_SC_–3T_SC_) for *R*_1_ (Δ*R*_1_, first row), *R*_2_ (Δ*R*_2_, second row), and AI (ΔAI, third row). Using binning strategy (1), WM voxels were binned into five FA intervals, that is (0.0, 0.2], (0.2, 0.4], (0.4, 0.6], (0.6, 0.8], and (0.8, 1.0], using θ bins spanning 0°–90° with a step size of 7.5°. Data represent mean ± standard deviation across all five matched subjects. (B) Representative axial field strength difference maps overlaid on the anatomical *T*_1_‐weighted dataset from one subject.

**FIGURE 4:**
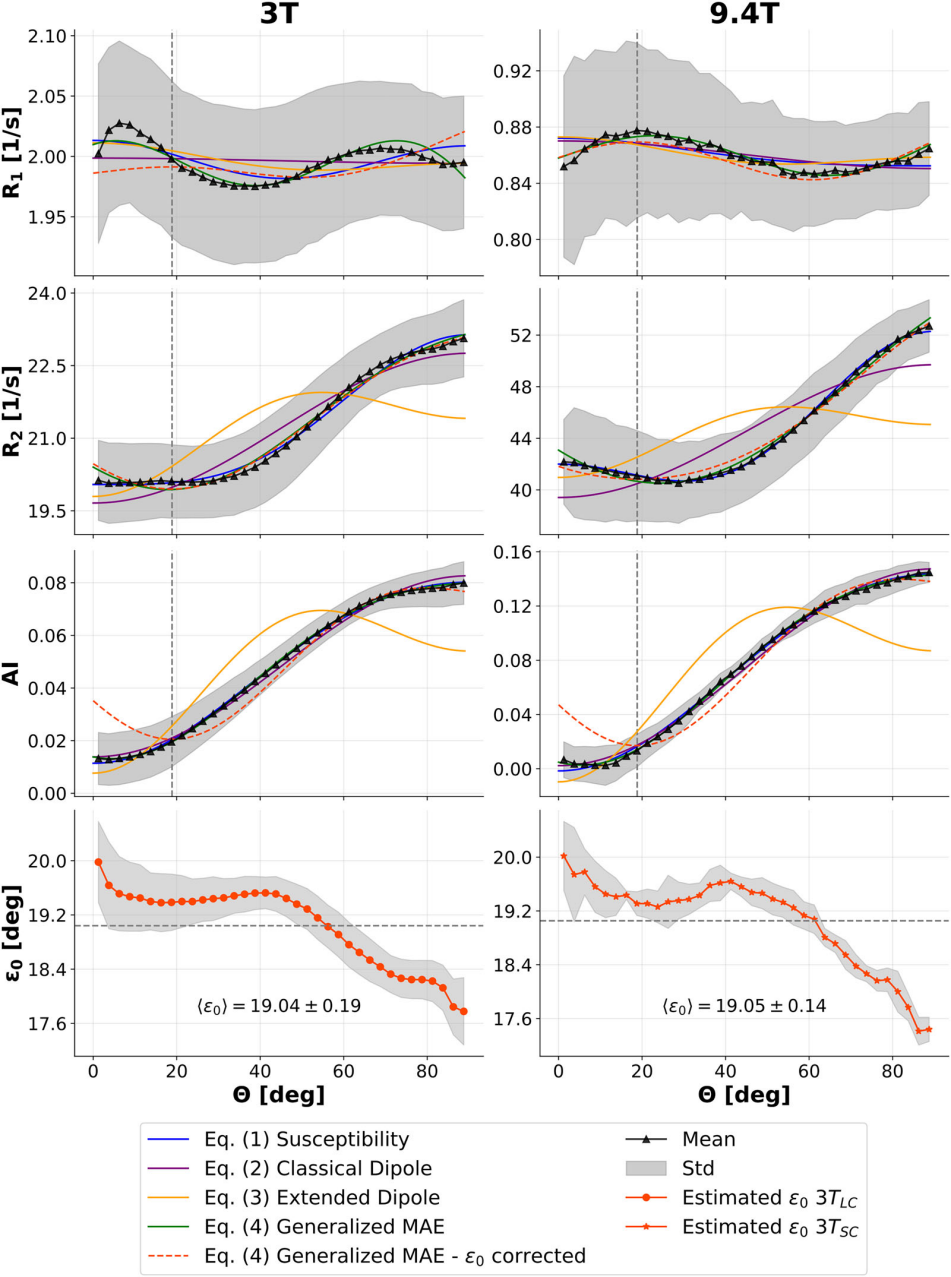
Model fitting of *R*_1_ (first row), *R*_2_ (second row), and AI (third row) anisotropy in white matter with high fractional anisotropy (0.5, 1.0]. Data from the large 3T cohort (left column) and small 9.4T cohort (right column) are displayed. Black triangles indicate mean values across subjects using binning strategy (2) (*θ* bins spanning 0°–90° with a step size of 2.5°), and shaded gray areas represent corresponding standard deviations. Fit parameters and goodness of fit (*R* ^2^) are summarized in Table 4. Proposed literature models for orientation‐dependent relaxation are fitted to the measured data, that is, susceptibility (Equation 1, blue), classical dipole–dipole interaction (Equation 2, purple), extended dipole–dipole interaction (Equation 3, yellow), generalized magic angle effect (Equation 4, green), and generalized magic angle effect with *ε*_0_ correction (Equation 4, orange) models. Each curve represents the model prediction as a function of the fiber‐to‐field angle (*θ*). The fitted curve for the ε_0_ corrected generalized magic angle model was shifted by the mean *ε*_0_ (gray dashed vertical (row 1–3) and horizontal (row 4) lines) to match the angle range of θ for comparability. The mean (orange) and standard deviation (gray) of *ε*_0_, estimated voxel‐wise from DTI, across all subjects as a function of θ is shown in the last row for 3T_LC_ (left) and 3T_SC_ (right). Note that no 9.4T DTI data was available.

The anisotropy in percentage of *R*_1_, *R*_2_, AI at 3 and 9.4T as well as of the corresponding field strength difference maps is summarized in Table 3 for high FA (0.5–1.0) WM bins (binning strategy (2)). Corresponding curves underlying the anisotropy calculation are shown in Figure 4 (3T_LC_, left column and 9.4T_SC_, right column). As evident from Table 3, the inter‐subject mean anisotropy increases from 3T (LC and SC) to 9.4T (SC) by about a factor two for all reported parameters. Specifically, *R*_2_ (≈7% at 3T, ≈13% at 9.4T) and AI (≈7%−8% at 3T, ≈14% at 9.4T) exhibit high and comparable values. The anisotropy of the field strength difference maps is visualized in Figure 3, revealing a clear orientation dependence for *R*_2_ and AI parameters in anisotropic WM (left column), with mean inter‐subject anisotropies at high FA (0.5–1.0) of ≈18% and ≈6%, respectively (cf. Table 3), and spatial patterns reflecting the underlying WM fiber architecture (right column).

**TABLE 3:** Anisotropies in percentage of *R*_1_, *R*_2_, and AI for the large cohort (3T_LC_, *n* = 107) and the small cohort (3T_SC_/9.4T_SC_, *n* = 5) as well as the anisotropies of the field strength differences of all three parameters for the subject‐matched cohort (9.4T_SC_–3T_SC_).

Parameter	Dataset	Mean ± std. (%)	Median (%)	Min (%)	Max (%)
*R*_1_	3T_LC_	2.25 ± 0.61	2.22	0.81	4.22
	3T_SC_	2.71 ± 0.73	2.95	1.34	3.45
	9.4T_SC_	4.25 ± 0.72	4.19	3.22	5.18
	9.4T_SC_–3T_SC_	−5.46 ± 2.21	−4.91	−9.14	−2.33
*R*_2_	3T_LC_	7.81 ± 0.93	8.03	5.21	9.94
	3T_SC_	7.73 ± 0.61	7.95	6.79	8.33
	9.4T_SC_	12.94 ± 1.92	13.01	10.78	16.29
	9.4T_SC_–3T_SC_	18.16 ± 4.11	17.72	13.88	25.85
AI	3T_LC_	6.93 ± 1.40	7.18	3.33	9.43
	3T_SC_	8.28 ± 1.23	8.44	6.04	9.39
	9.4T_SC_	14.15 ± 2.09	14.20	10.61	17.09
	9.4T_SC_–3T_SC_	6.28 ± 1.07	5.98	4.91	8.19

*Note*: The inter‐subject mean ± standard deviation, median, minimum, and maximum of the anisotropy are displayed. The anisotropy calculation is described in Section 2.4.

Figure 4 and Table 4 present the model fitting results of *R*_1_, *R*_2_, and AI at 3T_LC_ and 9.4T_SC_ for high FA (0.5–1.0) WM bins. For *R*_2_ and AI, the susceptibility and generalized MAE models achieve equally high goodness of fit ($R^{2}\geq 0.99$) at both field strengths. *R*_1_ demonstrates generally lower explainability by the employed models, with the generalized MAE model using *ε*_0_ as free fitting parameter performing best ($R^{2}=0.82$ for 3T_LC_ and 0.92 for 9.4T_SC_). The *R*_2_ fit with the susceptibility model yields a stronger weighting of the sin^4^ *θ* term as compared to the sin^2^ *θ* term at both field strengths (in particular at 3T). Conversely, the AI fit with the susceptibility model yields a stronger weighting of the sin^2^ *θ* term at both field strengths. The classical dipole model fits AI accurately ($R^{2}\geq 0.99$) at both field strengths while the extended dipole model fails to describe any of the fitted data properly. In agreement with literature, the generalized MAE fits of *R*_2_ yield *α* estimates of approximately 70° for both 3T_LC_ and 9.4T_SC_ and *ε*_0_ estimates of 17.99° (3T_LC_) and 24.27° (9.4T_SC_) [47, 48, 49]. Interestingly, at 3T the fitted and mean measured *ε*_0_ values are closer for the 3T_LC_ data (fitted *ε*_0_ = 17.99°, mean measured *ε*_0_ = 19.04°) than for the 3T_SC_ data (fitted *ε*_0_ = 30.06°, mean measured *ε*_0_ = 19.05°). Voxel‐wise analysis of data corrected by *ε*_0_ shows reproducibility in all parameters compared to model fits with *ε*_0_ as a free fitting parameter, but also reveals overall reduced *R*_2_ values, especially for *R*_1_ and AI at both field strengths. The fitted anisotropic *R*_2_ relaxation component $\left(R_{2,a}\right)$ is clearly higher at 9.4T as compared to 3T, yielding $R_{2,a}\left(9.4T_{\mathrm{SC}}\right)/R_{2,a}\left(3T_{\mathrm{SC}}\right)=3.3$ (subject‐matched) and $R_{2,a}\left(9.4T_{\mathrm{SC}}\right)/R_{2,a}\left(3T_{\mathrm{LC}}\right)=4.7$ (not subject‐matched). Based on the fitted $R_{2,a}$ values, the estimated partial susceptibility contribution to $R_{2,a}$ increases from 39.0% at 3T to 87.1% at 9.4T by comparing 3T_LC_ and 9.4T_SC_ datasets, and from 24.0% at 3T to 77.0% at 9.4T by comparing the subject‐matched 3T_SC_ and 9.4T_SC_ datasets.

**TABLE 4:** Model fitting results for *R*_1_, *R*_2_, and AI versus fiber‐to‐field angle for high FA (0.5–1.0) WM bins in the 3T large‐cohort (3T_LC_, *n* = 107) and 9.4T small‐cohort (9.4T_SC_, *n* = 6) data, shown in Figure 4.

		*R*_1_		*R*_2_		AI	
Model	Parameter	3T_LC_	9.4T_SC_	3T_LC_	9.4T_SC_	3T_LC_	9.4T_SC_
Susceptibility	*a*	2.01	0.87	20.04	42.00	0.01	0.00
	*b*	−0.12	−0.04	0.03	−10.43	0.09	0.18
	*c*	0.12	0.02	3.07	20.72	−0.02	−0.03
	*R* ^2^	0.52	0.50	0.99*	1.00*	1.00*	1.00*
Classical dipole	*a*	2.00	0.86	21.72	46.27	0.06	0.10
	*b*	0.00	0.01	−1.03	−3.43	−0.02	−0.05
	*R* ^2^	0.01	0.48	0.93	0.80	0.99	1.00*
Extended Dipole	*a*	1.99	0.85	21.95	46.44	0.07	0.12
	*b*	0.01	0.00	−0.54	−1.37	−0.02	−0.03
	*R* ^2^	0.26	0.41	0.40	0.20	0.71	0.69
Generalized MAE	*p*_i_	1.73	0.83	17.64 (16.63)^a^	28.32	−0.03	−0.09
	*p*_a_	0.24	0.04	5.99 (8.58)^a^	28.30	0.12	0.25
	*α*	35.26	90.00	69.15 (70.20)^a^	70.23	68.90	68.90
	*ε*_0_	39.05	22.86	17.99 (30.06)^a^	24.27	4.56	5.64
	*R* ^2^	0.82*	0.92*	0.99* (0.99*)^a^	1.00*	1.00*	1.00*
Generalized MAE—*ε*_0_ corrected	*p*_i_	1.94	0.83	17.75 (16.82)^a^	28.86	0.00	−0.04
	*p*_a_	0.09	0.04	5.83 (7.41)^a^	26.03	0.09	0.19
	*α*	74.56	90	68.97 (71.78)^a^	70.88	66.05	66.46
	*R* ^2^	0.51	0.84	0.99* (0.97)^a^	0.99	0.94	0.95

*Note*: Five models were fitted: (1) susceptibility [42] (Equation 1), (2) classical dipole [75, 76] (Equation 2), (3) extended dipole [40, 73, 77] (Equation 3), (4) generalized magic angle effect [47, 48, 49] (Equations 4 and 5), (5) generalized magic angle effect with voxel‐wise *ε*_0_ correction using DTI‐based estimates, eliminating *ε*_0_ as free fitting parameter. *R* ^2^ (coefficient of determination) indicates the goodness of fit. The best fitting model (according to *R* ^2^) for each parameter is indicated by a asterisk (*).

^a^ Values in brackets indicate *R*_2_ estimates obtained from generalized MAE model fitting for the 3T small‐cohort data (3T_SC_, *n* = 5).

**FIGURE 5:**
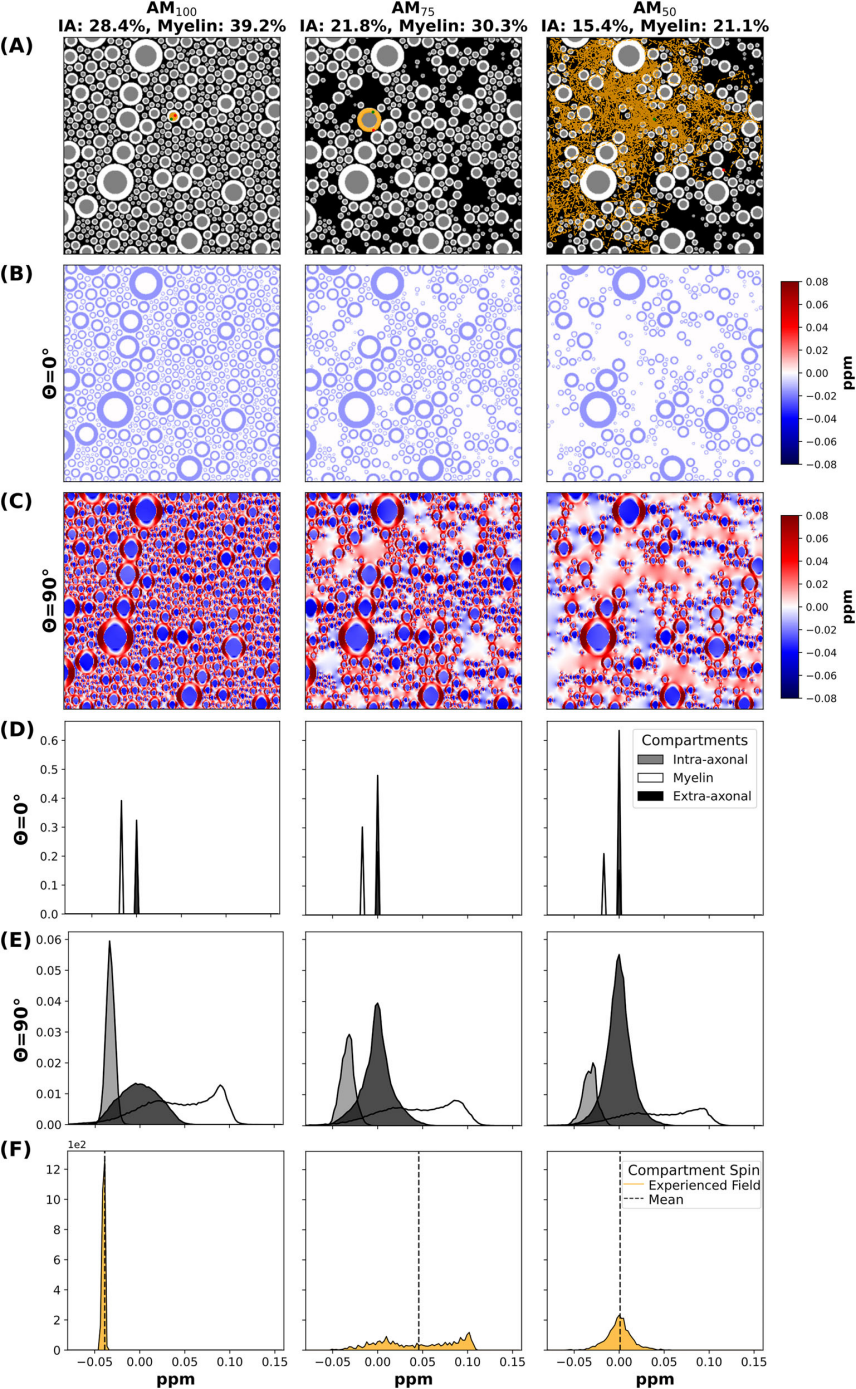
White matter axon models and corresponding mesoscopic magnetic field perturbations used as input for the Monte Carlo simulations with SpinWalk. (A) Axon packing models with varying dropout levels (AM_100_, AM_75_, AM_50_). (B, C) 2D magnetic field perturbations at fiber‐to‐field angles of *θ* = 0° (B) and *θ* = 90° (C). The WM tissue masks (A) display representative random walk trajectories (orange lines) sampled every 1000 steps for a single spin within different compartments (AM_100_: Intra‐axonal, AM_75_: Myelin, AM_50_: Extra‐axonal), with green and red markers indicating trajectory start and end points, respectively. (D, E) Orientation‐dependent frequency distributions of each compartment corresponding to *θ* = 0° (D) and *θ* = 90° (E). (F) Distribution of frequencies experienced by the spins indicated in orange in (A) during their random walks.

Axon packing model variants (AM_100_, AM_75_, AM_50_) used for the Monte Carlo simulations with SpinWalk are illustrated in Figure 5 alongside their microstructural field characteristics. Figure 5A presents cross‐sectional masks with intra‐axonal and myelin volume fractions decreasing systematically with increasing dropouts, alongside selected representative spin trajectories of every 1000 steps (orange) in the three different compartments. Figure 5B,C display susceptibility‐induced field maps at θ=0° and θ=90°, revealing orientation‐dependent shifts and increased spatial heterogeneity with reduced packing density. Figure 5D,E present the respective compartment‐resolved frequency distributions for θ=0° (D) and θ=90° (E). Frequency peaks are distinct at θ=0° and broaden at θ=90°. Figure 5F shows the frequency offsets sampled during the representative random‐walk spin trajectories indicated in Figure 5A.

**FIGURE 6:**
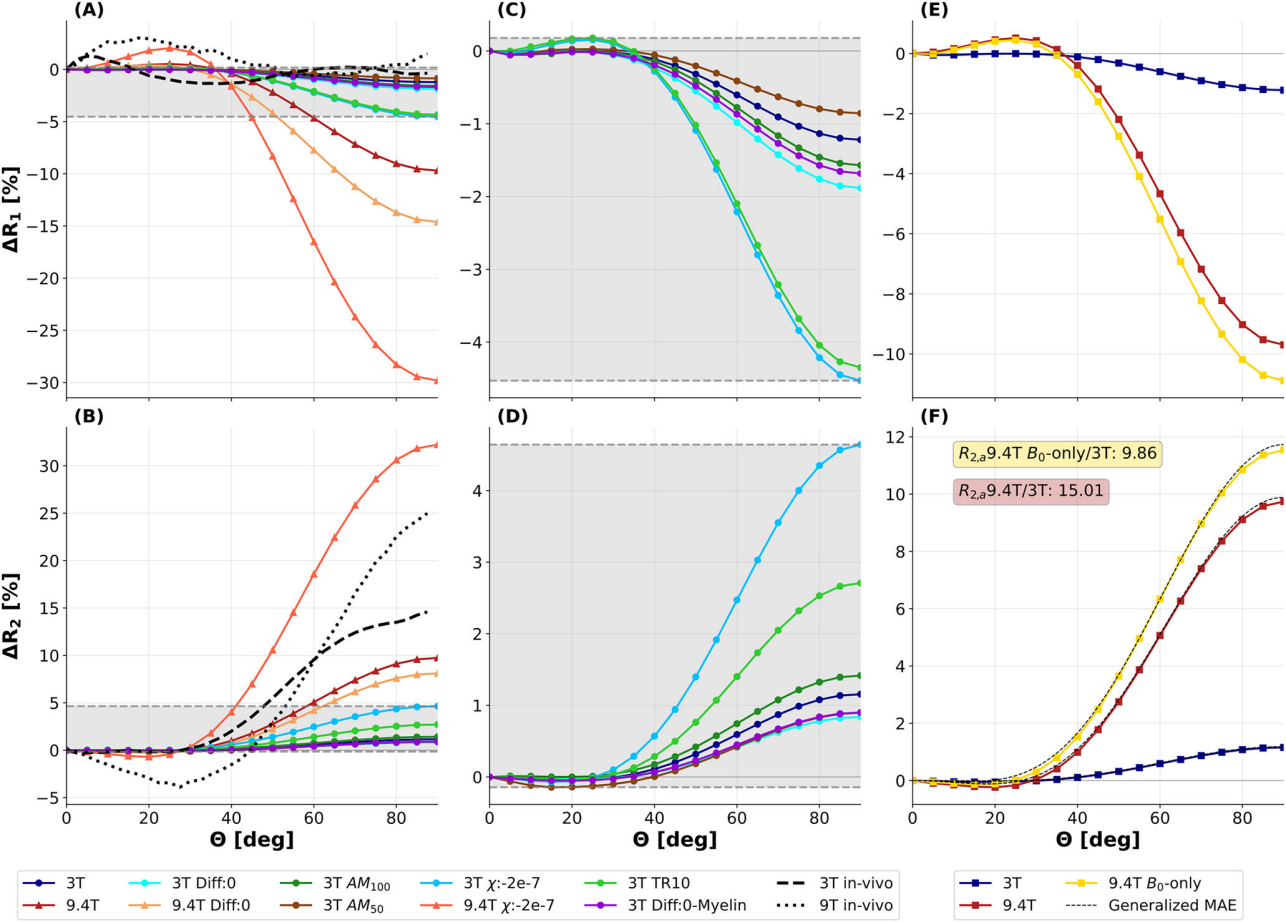
SpinWalk simulation results using the combined signal from all compartments for relaxometry. The orientation dependence of *R*_1_ (first row) and *R*_2_ (second row) is plotted as a function of the fiber‐to‐field angle (*θ*) relative to *θ* = 0° in percentage. (A, B) Plots for different 3 and 9.4T SpinWalk configurations (cf. Table 2). The in vivo results are added for direct comparison (large‐cohort 3T: Black dashed, small‐cohort 9.4T: Black dotted). (C, D) Plots of 3T SpinWalk configurations only. The gray shaded area indicates the same *y*‐axis range as the shaded area in subplots (A, B). (E, F) Generalized MAE model (cf. Equation 4) fitted to the default SpinWalk configurations at 3T (dark blue) and at 9.4T (red) as well as to a 9.4T SpinWalk configuration where relaxation rates and pc‐bSSFP parameters were matched to the default 3T configuration to isolate the effect of *B*_0_ (yellow). The corresponding fitted $R_{2,a}$ ratios are displayed in text boxes.

Relaxation anisotropy based on different 3 and 9.4T SpinWalk configurations (cf. Table 2) is assessed in Figure 6 versus the fiber‐to‐field angle, demonstrating that the orientation dependence is clearly enhanced at 9.4T as compared to 3T. *R*_1_ and *R*_2_ exhibit similar, but opposing, anisotropy characteristics, with *R*_1_ decreasing (cf. Figures 6A,C) and *R*_2_ increasing (cf. Figure 6B,D) relative to θ=0° for higher fiber‐to‐field angles. While the observed in silico anisotropy pattern of *R*_2_ resembles the in vivo data (dashed and dotted black curves in Figure 6A,B), although appearing underestimated, the in silico *R*_1_ anisotropy fails to reproduce the in vivo observations completely. The *R*_1_ and *R*_2_ anisotropy appears strongly influenced by the susceptibility difference Δ*χ* between myelin and intra‐/extra‐axonal water (cf. cyan (3T) and orange (9.4T) curves simulated with doubled Δ*χ*) as well as by the choice of the repetition time, which determines the periodicity (=1/TR) of the bSSFP frequency response (cf. light green (3T) curve simulated with doubled TR). On the other hand, the impact of different axon packing models (AM_100_ vs. AM_50_) remains rather weak. Interestingly, reduced diffusion (either setting the diffusion coefficient to 0 for all compartments or for the myelin compartment only) decreases the orientation dependence of *R*_2_, but increases the one of *R*_1_. The fitted ratio $R_{2,a}\left(9.4\;T\right)/R_{2,a}\left(3\;T\right)$ for SpinWalk simulations, which differed solely in *B*_0_, was found to be 9.86, thus close to the nominal ratio (η=10.56), as expected since SpinWalk is based on a susceptibility model. Representative bSSFP profiles generated with SpinWalk are included in Figure S3 in comparison to the in vivo profiles for reference.

## 4 Discussion

This work characterizes field strength‐dependent WM *R*_1_, *R*_2_, and AI anisotropy jointly derived based on pc‐bSSFP experiments at 3 and 9.4T, both in vivo and in silico.

### 4.1 *R*_1_ Anisotropy

The rather weak angular *R*_1_ anisotropy in WM reported in literature varies from about 1.2% to 4.9% [50, 52, 53, 54, 55], thus in the same range as our observations (cf. Table 3). In the mentioned studies, the observed orientation dependence of *R*_1_, which exhibited either a magic angle effect (minimum of *R*_1_ near the magic angle) [52, 54, 55] or a monotonic increase of *R*_1_ with *θ* [50, 53], has been primarily explained by anisotropic dipole–dipole interactions of water molecules near myelin lipid bilayers. Here, *R*_1_ shows an oscillating behavior versus *θ* in WM (cf. Figure 4), which could not be replicated by the susceptibility model implemented with SpinWalk (cf. Figure 6). This suggests the involvement of additional biophysical mechanisms beyond susceptibility effects such as dipole–dipole interactions [50, 53], aligning with prior work arguing against magnetic susceptibility of myelin as the dominant source of *R*_1_ orientation dependence [51, 52, 53, 54, 55]. The variability of reported *R*_1_ anisotropy patterns may be attributed to the variety of different sequences employed and their different sensitivities to magnetization transfer or B_1_^+^ inhomogeneity, constituting two main factors influencing *R*_1_ quantification (inversion recovery, MP2RAGE, variable flip angle in previous studies [50, 52, 53, 54, 55], pc‐bSSFP in this study).

### 4.2 *R*_2_ Anisotropy

The angular anisotropy of *R*_2_ in anisotropic WM structures reported in literature consistently points to a pronounced increase of *R*_2_ with higher fiber‐to‐field angles similar as observed for *R*_2_* [41, 45, 47, 49, 98, 99], in alignment with our findings (cf. Figures 2, 3, 4).

Different theories have been proposed to explain the biophysical mechanisms underlying *R*_2_ anisotropy. In the context of spin‐echo *R*_2_ relaxation, the observed orientation dependence [41] was traced back to diffusion‐mediated dephasing due to susceptibility differences induced by the myelin sheath (sin^4^θ term in Equation 1) as potential main contributor [42, 99]. In recent work, a generalized MAE model (Equation 4), which assigns orientation dependence to non‐averaging proton RDC in heterogeneous microenvironments [47], was suggested as possible source of WM *R*_2_ anisotropy.

The susceptibility model (Equation 1) and both generalized MAE models, with and without treating *ε*_0_ as free fitting parameter (Equation 4), fitted the orientation dependence of *R*_2_ in WM accurately for our data ($R^{2}\geq 0.99$ at both field strengths). The ambiguity of those models (Equation 1 would become mathematically equivalent to Equation (4) if the offset angle *ε*_0_ was included as well ($\theta \to \theta -\varepsilon_{0}$), even though with differently parametrized fitting variables [47]) challenges a direct disentangling of either susceptibility or dipole–dipole sources. However, the field strength dependence of *R*_2_, observed in this work, enables novel insights into the driving mechanisms as outlined in the following.

The *R*_2_ field strength difference maps exhibit a pronounced fiber‐to‐field angle sensitivity (cf. Figure 3 and anisotropy values in Table 3), indicating a contribution of susceptibility effects. The ratio of the fitted anisotropic *R*_2_ components, that is, $R_{2,a}\left(9.4\;T\right)/R_{2,a}\left(3\;T\right)$ was found to be in the range of 3.3–4.7, pointing to multiple sources of *R*_2_ anisotropy as the case $R_{2,a}\left(9.4\;T\right)/R_{2,a}\left(3\;T\right)=1$ indicates no change in $R_{2,a}$ with field strength increases and thus pure magic angle effects, while $R_{2,a}\left(9.4\;T\right)/R_{2,a}\left(3\;T\right)=10.56=\eta$ indicates pure susceptibility effects. The partial susceptibility contribution estimated based on the fitted $R_{2,a}$ values (24.0%–39.0% at 3T versus 77.0%–87.1% at 9.4T) suggests that *R*_2_ anisotropy at 9.4T may be dominated by susceptibility mechanisms whereas additional sources such as residual dipolar couplings likely contribute at 3T. To further validate the fit of the generalized MAE model and anisotropic $R_{2,a}$ (Equation 4), we estimated the offset angle *ε*_0_ directly from DTI‐derived axial and radial diffusivity [47, 48] (cf. last row Figure 4) and repeated the fitting without treating *ε*_0_ as a free fitting variable, yielding similar partial susceptibility contributions (26.3%–36.3% at 3T vs. 79.0%–85.7% at 9.4T). In a recent study, only slightly increased *R*_2_ anisotropy at 3T as compared to 1.5T were reported [98], which seems consistent with our findings since the partial contribution of susceptibility may be further reduced at lower field strengths.

The susceptibility‐based SpinWalk simulations, which could reproduce the *R*_2_ anisotropy pattern, even though not its extent, corroborate the influence of mesoscopic field perturbations due to the magnetic susceptibility of myelin. A compartmental analysis in Figure S4 (3T) and Figure S5 (9.4T), demonstrates that myelin *R*_2_ clearly exhibits the strongest angular anisotropy, followed by the extra‐axonal *R*_2_, while the intra‐axonal *R*_2_ shows the weakest orientation dependence in agreement with previous work [43]. Anisotropy in the combined three‐compartment signal is thus expected to increase with the weighting (volume fraction times proton density) of the myelin component as confirmed by the SpinWalk simulations provided in Figure S6.

The simulated microscopic diffusion of water molecules affects the resulting *R*_2_ relaxation anisotropy only slightly as compared to the static case without any diffusion (cf. Figure 6). Simulating *R*_2_ at a fiber‐to‐field angle of 90° versus diffusion coefficient (cf. Figure S7) indicates that the default diffusion coefficients utilized in the simulations (dashed vertical line in Figure S7) may lie in the motional narrowing regime. In this regime, the spin dephasing effect of diffusion on *R*_2_ is reduced since all spins accumulate similar frequency offsets.

Interestingly, we observe that the fit of Equation (1) leads to a 100 × stronger weighting of the sin^4^ *θ* term (c=3.07) as compared to the sin^2^θ term (b=0.03) at 3T, while the weighting of sin^4^ *θ* and sin^2^ *θ* is becoming comparable at 9.4T (cf. Table 4). Since bSSFP may undergo spin‐echo like refocusing at TE=TR/2 as long as the intra‐voxel frequency dispersion is smaller than ±1/(2TR) [100, 101], which may be more easily fulfilled at 3T as compared to ultra‐high fields, we could argue that the anisotropy of pc‐bSSFP‐derived *R*_2_ at 3T must follow similar characteristics like spin‐echo *R*_2_, while at 9.4T it may approach characteristics like gradient‐echo *R*_2_*, commonly reported to be driven by a sin^2^ *θ* dependence arising from the typical assumption of a hollow‐cylinder model of susceptibility differences (as also used in this work for the SpinWalk simulations).

All these considerations point to mesoscopic susceptibility effects as likely factors affecting pc‐bSSFP *R*_2_ anisotropy, with an increased contribution at higher field strength. Thereby, mesoscopic susceptibility heterogeneities can arise from multiple sources such as the anisotropic magnetic susceptibility of myelin sheaths, spatial variations in myelin density within voxels, and susceptibility discontinuities at boundaries between regions of different fiber orientations.

### 4.3 Asymmetry Index

The bSSFP frequency response is directly modulated by the underlying frequency content in a voxel. Asymmetries of the intra‐voxel frequency distribution can be visualized voxel‐wise by the asymmetry index AI. In case of white matter, the AI directly reflects tissue microstructure such as the orientation of anisotropic WM fiber tracts relative to *B*_0_ (≈0 at θ=0° and maximal at θ=90°), as observed in this work and reported previously [9, 10, 13, 14]. Similarly to *R*_2_, the AI demonstrates a strong field strength dependence (cf. Figure 3 and Table 3), corroborating the influence of susceptibility. The assumption of a susceptibility‐shifted myelin compartment introduces likely a periodic dependence of AI (and possibly *R*_2_) on the repetition time (TR) with a cycle of 1/($\bar{\gamma }∆\chi B_{0}$) [10]. However, since the susceptibility shift of myelin is low (∆χ≈0.1ppm, leading to periodicities of about 81 and 25 ms at 3 and 9.4T, respectively), it is difficult to probe this in practice.

The orientation and field strength dependence of the asymmetry index point to mesoscopic susceptibility effects introduced by the structural anisotropy of the axons and the susceptibility anisotropy of the lipid molecules within the myelin sheath as main contributing factors of bSSFP profile asymmetries in WM. However, other sources involving the presence of molecular species with different chemical or susceptibility shifts (e.g., lipids, proteins, iron‐bearing molecules, deoxyhemoglobin) can potentially contribute to asymmetries in the intra‐voxel frequency distribution and thus bSSFP frequency response. Chemical exchange can cause bSSFP profile asymmetries as well, but its influence was reported to be negligible in WM [10].

### 4.4 Limitations

Several methodological considerations must be acknowledged when interpreting the observed orientation‐dependent relaxation anisotropy. Anisotropic magnetic susceptibility of myelin relative to intra‐ and extra‐axonal water generates orientation‐dependent microscopic field inhomogeneities that scale with *B*_0_. These susceptibility‐induced spatially varying field gradients directly modulate intra‐voxel spin dephasing, creating intrinsic orientation‐dependent transverse relaxation. However, these same field gradients may simultaneously introduce asymmetries in bSSFP frequency profiles, particularly when multiple compartments with different frequency offsets coexist within a voxel. The MIRACLE fitting algorithm, which is based on analytical signal models assuming symmetric single‐compartment profiles, which are characterized by single‐component relaxation and a single localized resonance frequency, becomes sensitive to these profile asymmetries. Importantly, both the genuine microstructural relaxation anisotropy and the fitting sensitivity originate from the same biophysical mechanism, that is, susceptibility‐induced microscopic field inhomogeneities from ordered myelin structures. The conducted Monte Carlo simulations demonstrate that even symmetric individual single‐compartment profiles can exhibit anisotropy due to spatially varying local field perturbations (cf. Figures S4 and S5), indicating that the observed in vivo relaxation anisotropy may reflect susceptibility‐driven dephasing.

Dipolar interactions in the partially ordered lipid bilayers of myelin can lead to an orientation dependence of magnetization transfer parameters [102] or result in orientation‐dependent non‐averaging proton residual dipolar couplings (RDCs) [47] in human brain WM. These effects are not accounted for by the susceptibility‐diffusion model explored in this work, which simulates the random walk of spins in inhomogeneous magnetic fields. The stronger orientation dependence observed in vivo as compared to in silico suggests that additional mechanisms including residual dipolar couplings or magnetization transfer may contribute. The employed three‐compartment WM model is further limited by treating compartments as impermeable. However, since the myelin water lifetime is estimated to range between 13 and 150 ms [103, 104], which is considerably longer than the bSSFP repetition times used in this work, the influence of compartmental exchange is likely small, but may increase for smaller axons with thinner myelin layers.

The relatively long duration of the 10‐min pc‐bSSFP acquisition could potentially lead to the accumulation of frequency offsets within a voxel due to head movement or *B*_0_ drift, which may bias the relaxation anisotropy analysis. Involuntary head motion could alter fiber‐to‐field angles, but due to the rather small rotations related to head movement of only few degrees as compared to the large natural variation in fiber orientation across the brain, we consider this effect to be minor. *B*_0_ drift over the course of the pc‐bSSFP scan is not expected to adversely affect the quantification of bSSFP profile asymmetries due to the employed robust metric (difference between signal peaks at positive and negative frequency offsets), but it could bias the relaxometry results [105]. By inspecting the first and last acquired bSSFP phase‐cycles, *B*_0_ drift was observed to be minimal over the course of the pc‐bSSFP acquisition at both 3 and 9.4T, in particular considerably smaller than the spacing used for sampling the bSSFP frequency response (i.e., ≪ 17.4 Hz at 3T/TR = 4.8 ms, ≪ 20.8 Hz at 9.4T/TR = 4 ms). *B*_0_ drift correction strategies exist for pc‐bSSFP relaxometry, for example, by assuming a linear drift [105], but their benefit may be reduced in case of small sub‐sample drifts as observed here due to the risk of introducing interpolation errors. The bias due to *B*_0_ drift in pc‐bSSFP relaxometry in the human brain, reported in a previous study, was relatively small (1%–5%), especially in comparison to the bias introduced by the presence of multiple components (about 30% underestimation) [105].

The employed binning strategy of global WM has the disadvantage that it might mix different structures with different tissue properties and potentially predominant fiber directions. Anatomical factors such as mean axon diameter, which is for example larger in the superior–inferior oriented corticospinal tract than in anterior–posterior oriented association fibers, may bias the anisotropy analysis, suggesting measurements with different head orientations, for example, using a tiltable RF coil [43]. To exclude anatomical variability as primary source of the observed anisotropies, we did an additional evaluation in five equidistant corpus callosum segments of varying fiber‐to‐field angles and fractional anisotropy (cf. Figure S8). The results confirm the overall trends seen in Figures 2 and S2, even though the full anisotropy patterns of *R*_1_, *R*_2_, and AI could not be reproduced due to the relatively narrow *θ* and FA ranges.

Our in vivo analysis assumes a single dominant fiber orientation per voxel, thus not considering crossing fibers or fiber dispersion, known to affect signal contrast [106] and shown to confound orientation‐dependent relaxation measurements [43, 98]. Given that 60%–90% of WM voxels contain crossing fibers [107, 108], further research is necessary to disentangle the effects of single versus crossing fibers, rather than just thresholding FA [98, 109].

The simulated field of view (43 × 43 × 43 μm^3^) in SpinWalk simulations was considerably smaller than typical in vivo voxels. Newer open‐source toolboxes (e.g., CACTUS [110]) can generate larger, more realistic fiber geometries, which could be incorporated in the future. Furthermore, modeling used exclusively parallel myelinated axons with the hollow cylinder fiber model, excluding crossing fibers and orientation dispersion effects like in vivo analysis.

## 5 Conclusion

This work characterizes for the first time pc‐bSSFP‐derived *R*_1_ and *R*_2_ anisotropy in human brain WM at 3 and 9.4T. Both *R*_2_ and AI show strong fiber‐to‐field angle dependence that scales with field strength. Model fitting and Monte Carlo spin‐walk simulations indicate that *R*_2_ anisotropy is influenced by the magnetic susceptibility of myelin, contributing substantially at 3T alongside other mechanisms like residual dipolar couplings, and becoming the primary driver at 9.4T with up to 87% susceptibility contribution. Beyond simultaneous *R*_1_ and *R*_2_ quantification, pc‐bSSFP provides field strength‐dependent AI maps—a third metric sensitive to tissue microstructure—and appears thus as a powerful tool to assess tissue integrity.

## Funding

This work was supported by Deutsche Forschungsgemeinschaft (HE 9297/1‐1) and European Research Council (834940).

## Supporting information


**Data S1:** mrm70255‐sup‐0001‐Supinfo.docx.

## Data Availability

The datasets analyzed during the current study are available from the corresponding author on reasonable request. The source code used to process the data and generate the figures will be available at https://github.com/s2quare/bssfp‐relaxation‐anisotropy.
