# Supplementary material for: Field Strength‐Dependent White Matter R 1 and R 2 Anisotropy of Phase‐Cycled Balanced Steady‐State Free Precession Relaxometry
Source: Magn Reson Med. 2026 Jan 23;95(5):2873–91. doi: 10.1002/mrm.70255 (PMC12962222; doi:10.1002/mrm.70255)
Supplement: Supplementary file 1 — Data S1: mrm70255‐sup‐0001‐Supinfo.docx. [file MRM-95-2873-s001.docx]

**Supporting Information**

**Field strength-dependent white matter R_1_ and R_2_ anisotropy of phase-cycled balanced steady-state free precession relaxometry**

**Correspondence to:**

Florian Birk, M.Sc.

High-Field Magnetic Resonance

Max Planck Institute for Biological Cybernetics

Max-Planck-Ring 11

72076 Tübingen, Germany

Email: florian.birk@tuebingen.mpg.de

Phone: +49 7071 601 719

Fax: +49-7071-601-702

White matter mask

For global white matter (WM) analysis, the mask was defined based on SynthSeg^1^ labels for left and right cerebral white matter combined with histogram analysis of R_1_ and R_2_ from both field strengths. For each subject, only voxels that were part of the SynthSeg labels and where R_1_ (3 T: [1.0, 3.0] 1/s, 9.4 T: [0.5, 1.5] 1/s) and R_2_ (3 T: [13, 28] 1/s, 9.4 T: [20, 69] 1/s) values fell within the boundaries at the respective field strength, or both field strengths in case of subject matched datasets, were included. The minimum and maximum relaxation rate boundaries were defined as the first quartile (Q1, 25th percentile) minus 1.5 times the interquartile range (IQR) and the third quartile (Q3, 75th percentile) plus 1.5 times the IQR, where IQR = Q3 – Q1, respectively. For R_1_ and R_2_, the boundaries were rounded to the nearest multiple of 0.5 1/s and the nearest integer value (1/s), respectively (see Supporting Information Figure S1).

Monte Carlo spin simulations

For phantom generation, 1000 axon disks were randomly initialized on a 2552×2552-pixel grid with 0.1×0.1 μm^2^ pixel resolution and subsequently packed towards the center of a 430×430-pixel region of interest (43×43 μm²) to maximize axon density while minimizing disk overlaps in 2D. Using the hollow-cylinder axon model^2^: Field perturbations resulting from susceptibility differences between myelin and intra-/extra-axonal compartments were calculated analytically on an axon-by-axon basis, incorporating both isotropic and anisotropic tensor components^2–4^:

$$\Delta B_{i}\left( r \right)=\left\{ \begin{aligned} \frac{\chi_{i}sin^{2}\theta cos2\Phi}{2}\left( \frac{r_{o}^{2}-r_{i}^{2}}{r^{2}} \right)\gamma&&r>r_{o} \\ \frac{\chi_{i}}{2}\left( cos^{2}\theta-\frac{1}{3}-sin^{2}\theta cos2\Phi\left( \frac{r_{i}^{2}}{r^{2}} \right) \right) \gamma&&r_{o} > r >r_{i} \\ 0&&r<r_{i} \end{aligned} \right.$$

$$\Delta B_{a}\left( r \right)=\left\{ \begin{aligned} \frac{\chi_{a}sin^{2}\theta cos2\Phi}{8}\left( \frac{r_{o}^{2}-r_{i}^{2}}{r^{2}} \right)\gamma& &r>r_{o} \\ \chi_{a}\left( {sin}^{2}\theta\left( -\frac{5}{12}- \frac{cos2\Phi}{8} \left( 1 + \frac{r_{i}^{2}}{r^{2}} \right) + \frac{3}{4}ln\left( \frac{r_{o}}{r} \right) \right)-\frac{{cos}^{2}\theta}{6} \right) \gamma&&r_{o} > r >r_{i} \\ \frac{3\chi_{a}sin^{2}\theta}{4}ln\left( \frac{r_{o}}{r_{i}} \right)\gamma&&r<r_{i} \end{aligned} \right.$$

where θ and $\Phi$ refer to the fiber-to-field angle and azimuthal angle of the susceptibility tensor, respectively. The radii r_i_ and r_o_ represent the inner and outer radii of the nested cylinder geometry. The magnetic susceptibility values for the isotropic and anisotropic tensor components of myelin are described by χ_i_ and χ_a_, respectively. The susceptibilities of the intra- and extra-axonal compartments were assumed to be zero. The total magnetic field perturbation was computed by combining the isotropic ($\Delta B_{i}$) and anisotropic ($\Delta B_{a}$) field components, which were derived under the assumption of χ_i_ = χ_a_, with the magnetic susceptibility of myelin relative to intra- and extra-axonal compartments set to –0.1 ppm unless stated otherwise^2–4^. The final field map was obtained by summing over all axons. Field map calculations were repeated for fiber orientations θ from 0° to 90° in 5° increments. The field maps, initially calculated for a 1 T reference field, were subsequently scaled within SpinWalk to correspond to the actual main magnetic field strength.

For Monte Carlo simulations of diffusing particles within the three-compartment (extra-axonal, EA; intra-axonal, IA; myelin) axon models during a pc-bSSFP experiment, the following default parameters were used: B₀ = 2.89 T / 9.39 T (nominal field strengths corresponding to the in vivo 3 T / 9.4 T data), diffusivity D_EA/IA_ = 1×10^-9^ m²/s, D_myelin_ = 0.1×10^-9^ m²/s^5–7^, T_1_ [EA, IA, Myelin] = [3 T: 1000, 1000, 300; 9.4 T: 1500, 1500, 400] ms, T_2_ [EA, IA, Myelin] = [3 T: 60, 60, 12; 9.4 T: 30, 30, 10] ms^8–13^, and 10⁵ spins. Sequence parameters (TR, TE, flip angle α_nom_, RF phase increments ϕ) were matched to the respective in vivo pc-bSSFP experiments at 3 T and 9.4 T (see Table 1 of the main manuscript). Dummy scans were set to (5×T_1_)/TR, where T_1_ represents the extra-axonal compartment relaxation time. The time step dt = 2 μs was selected such that the step length $r=\sqrt{\left( 6*D*dt \right)}$ remained smaller than half the radius of the smallest cylinder^14^. Eleven simulation configurations were performed and are shown in Table 1 of the main manuscript. For each configuration, Monte Carlo simulations were repeated for 19 fiber-to-field angles (θ) at 12 phase cycles to sample the bSSFP frequency response, requiring approximately 5–7.5 hours on a Tesla V100-PCIE-32GB GPU for one entire configuration simulation. In a final step the combined pc-bSSFP signal was calculated as $S_{combined}= \sum{WF}_{i} * S_{i}$**,** where ${WF}_{i} = ({VF}_{i} * {PD}_{i}) / \sum{VF}_{i} * {PD}_{i}$ represents the normalized water fractions based on volume fraction (VF) and proton density weighting (PD: EA=1.0, IA=1.0, myelin=0.5)^11,15,16^ and S_i_ the mean transverse magnetization in for compartment i (EA, IA, myelin). The myelin water fraction (MWF) was defined as $MWF = W_{myelin} * 100$, representing the weighted contribution of the myelin compartment to the total signal.

Additional details about the Monte Carlo simulations with SpinWalk regarding (1) the shape of the generated complex-valued pc-bSSFP data in comparison to the in vivo measurements and (2) the effect of myelin proton density weighting on orientation dependence are provided in Supporting Information Figures S6 and S7, respectively.

Corpus callosum analysis

To evaluate the orientation dependency of R_1_, R_2_, and AI in a single WM structure, exhibiting regionally different fiber-to-field angles, the corpus callosum (CC) was divided into five segments using an atlas-based approach for the large-cohort 3 T data. The analysis utilized a pre-defined DTI-based white matter atlas from Johns Hopkins University (JHU) containing an existing three-part CC (genu, callosal body, and splenium) segmentation in Montreal Neurological Institute (MNI) space^17^. To achieve finer anatomical resolution, the CC was further subdivided into five equidistant anterior-posterior segments, which approximately correspond to the genu (CC1), rostral body (CC2), mid-body (CC3), posterior body (CC4), and splenium (CC5). Manual segmentation was performed in MNI space using ITK-SNAP^18^. For subject-specific analysis, the native T_1_-weighted datasets were non-linearly registered to the MNI atlas, and the inverse transformations were subsequently applied to the CC segmentations, yielding CC segmentations in native T_1_-weighted space for each subject. This approach enabled the analysis of orientation-dependent effects in R_1_, R_2_, and AI across different CC segments (cf. Supporting Information Figure S8).


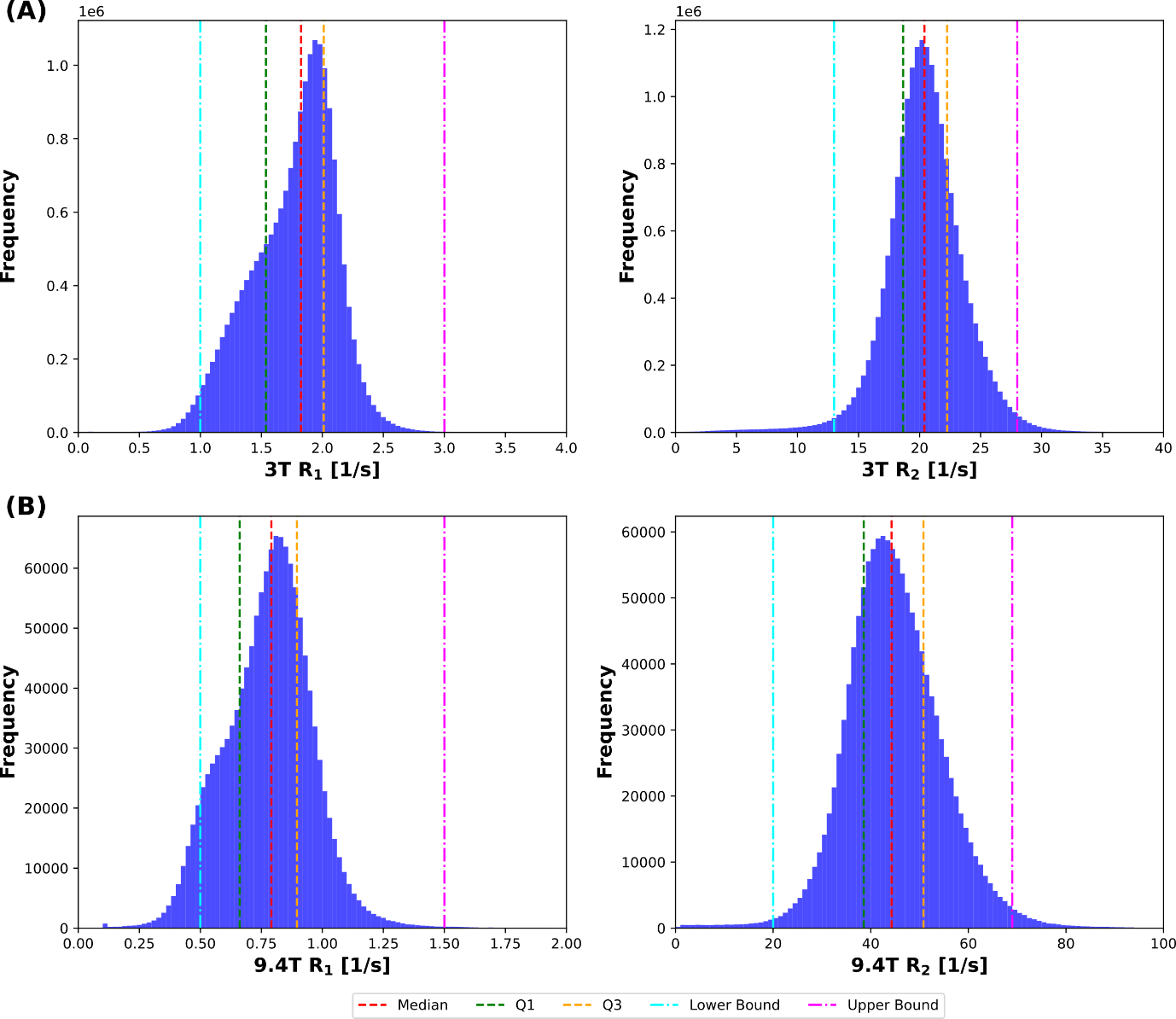


**Supporting Information Figure S1.**Whole-brain white matter (WM) histograms of R_1_ and R_2_ calculated based on SynthSeg WM labels. The distribution of relaxation rates in the large-cohort 3 T (**A**) and small-cohort 9.4 T (**B**) datasets were used to define inclusion boundaries for subsequent WM analyses. The median (red), first quartile (Q1, green), and third quartile (Q3, orange) are indicated by dashed lines. The lower (cyan) and upper (magenta) boundaries correspond to Q1 – 1.5 × IQR and Q3 + 1.5 × IQR, respectively, where IQR = Q3 – Q1. For practical implementation, R_1_ boundaries were rounded to the nearest multiple of 0.5 1/s and R_2_ boundaries to the nearest integer value (1/s). These boundaries were applied to exclude outlier voxels from the global WM analysis.

**
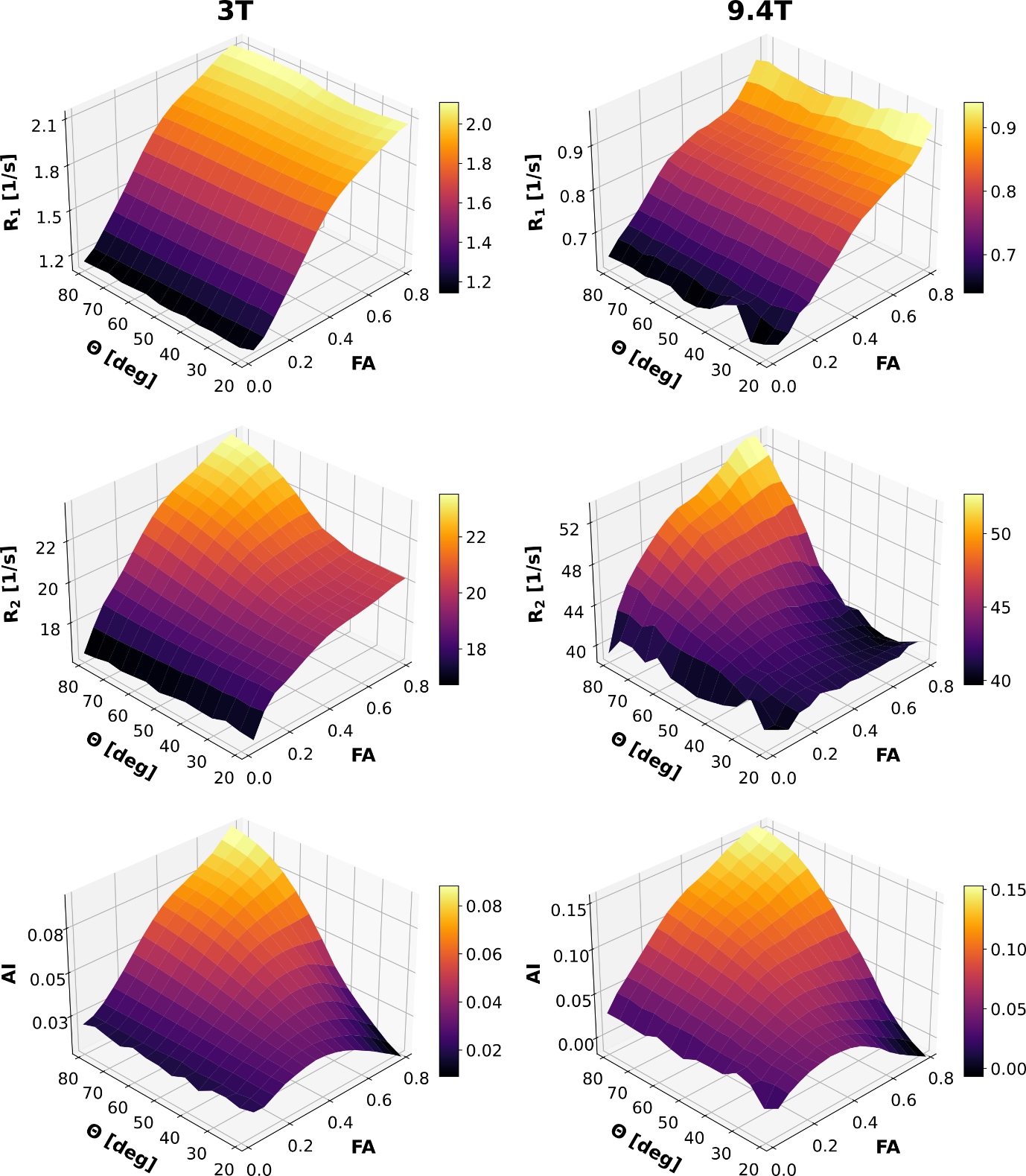
Supporting Information Figure S2.** 3D visualization of R_1_ (first row), R_2_ (second row), and AI (third row) anisotropy in global white matter versus fiber-to-field angle (θ) and fractional anisotropy (FA). Inter-subject mean values obtained from the large 3 T cohort (left column) and the small 9.4 T cohort (right column) are displayed. Data points represent mean values within FA (spanning 0.0–0.8 with a step size of 0.05) and θ (spanning 15°–90° with a step size of 5°) bins, corresponding to binning strategy (3).


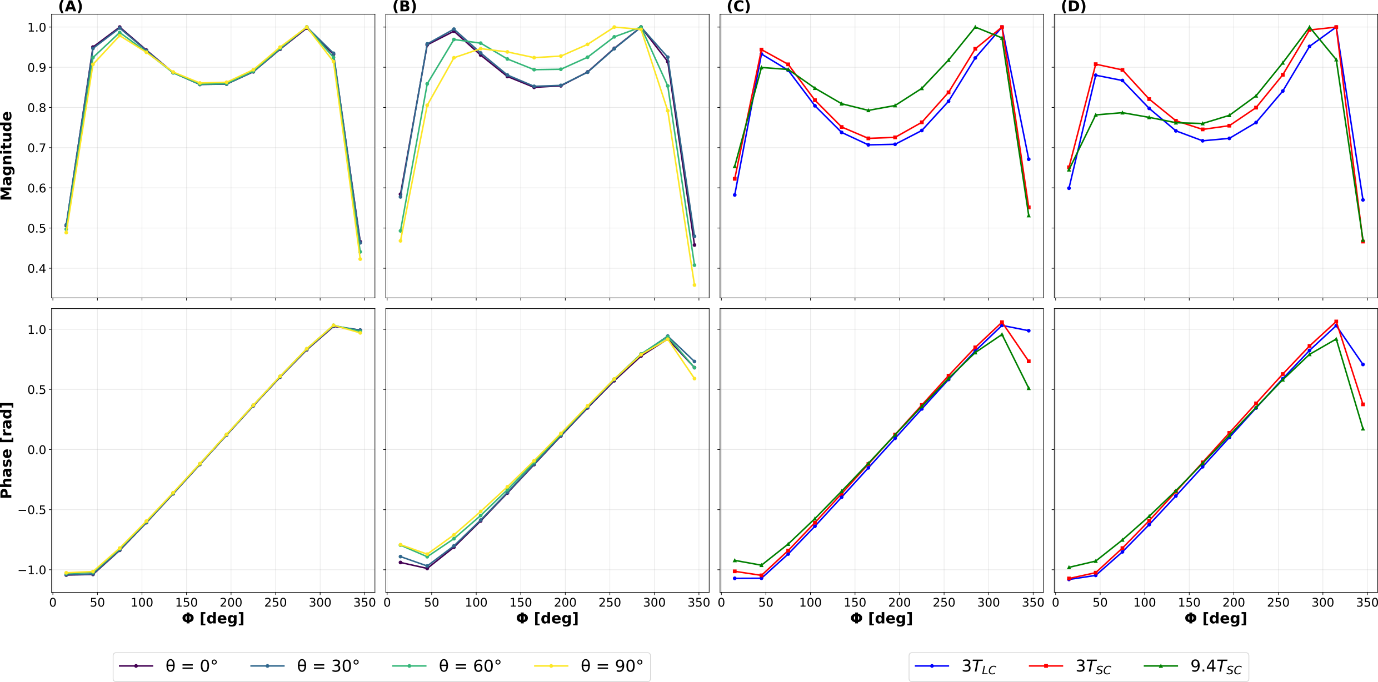


**Supporting Information Figure S3**. Comparison of pc-bSSFP frequency response profiles between SpinWalk simulations and in vivo data. Magnitude (top row) and phase (bottom row) of bSSFP frequency response profiles for SpinWalk simulations at 3 T (A) and 9.4 T (B) using Δχ = –0.2 ppm (see configuration 3T Δχ:-2e-7 and 9.4T Δχ:-2e-7 in Table 2 of the main manuscript), compared with white matter (WM) in vivo data separated by low (C, θ = (0°, 45°]) and high (D, θ = (45°, 90°]) fiber-to-field angles. The in vivo profiles represent cerebral WM data from representative subjects across three cohorts: 3T large-cohort (3T_LC_, blue), 3T small-cohort (3T_SC_, red), and 9.4T small-cohort (9.4T_SC_, green). WM regions were identified using SynthSeg segmentation with refined boundary conditions (detailed in the *White matter mask* section) and further restricted to areas with high fractional anisotropy (FA > 0.5). The complex-valued bSSFP profiles are B_0_-corrected and the magnitudes are normalized to their maximum value to enable direct comparison.


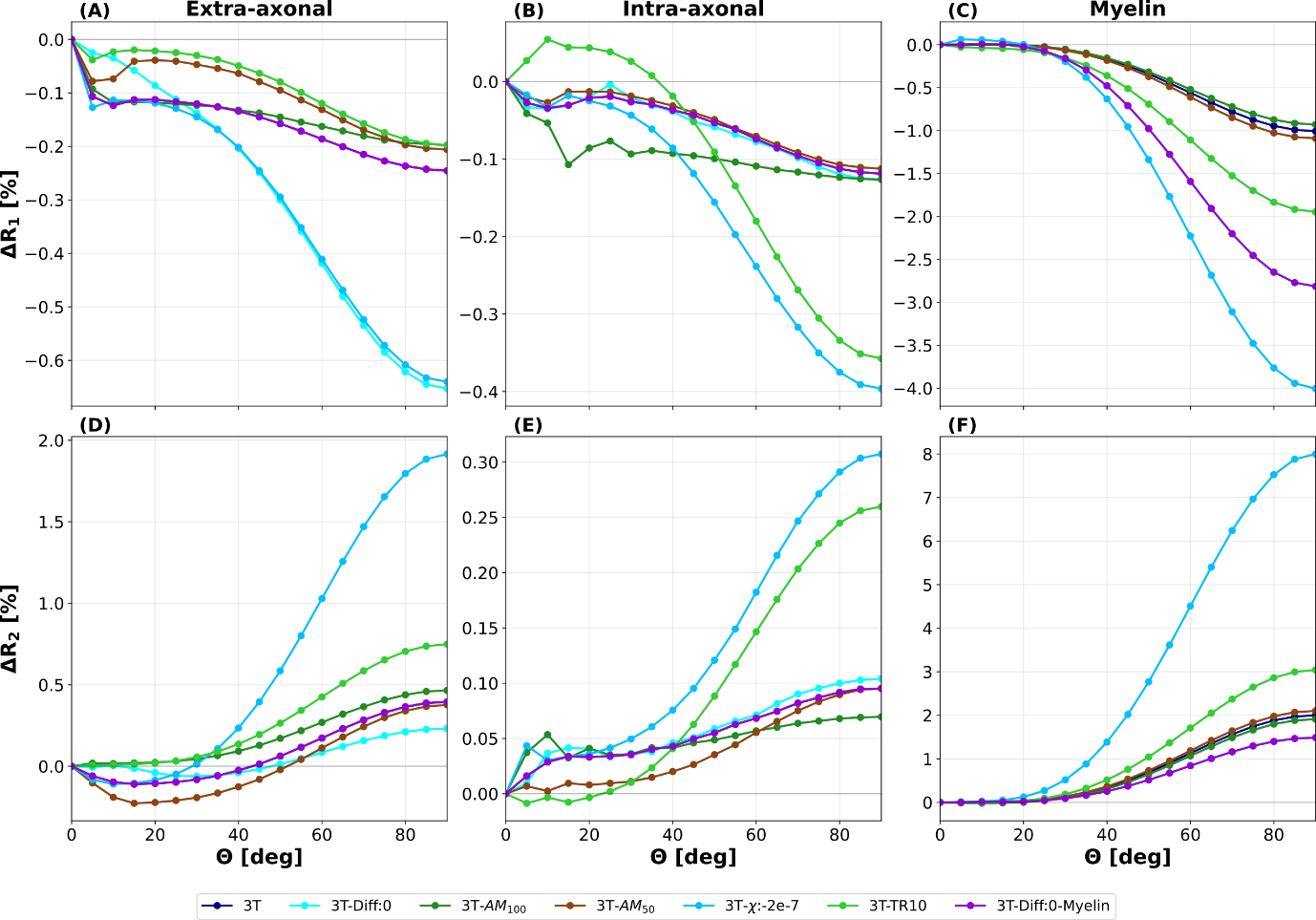


**Supporting Information Figure S4.** Compartmental analysis of SpinWalk simulations at 3 T. The relative orientation dependence of R_1_ (first row, **A-C**) and R_2_ (second row, **D-F**) is plotted as a function of fiber-to-field angle (θ) for the three individual compartments. Results are shown separately for extra-axonal (A, D), intra-axonal (B, E), and myelin (C, F) compartments. Different SpinWalk configurations are represented by distinct colors: default 3 T protocol (dark blue), no diffusion for all compartments (cyan), different axon models (dark green: AM_100_, dark brown: AM_50_), doubled magnetic susceptibility difference of myelin relative to intra-/extra-axonal water (light blue), altered repetition and echo times (green: TR/TE = 10/5 ms), and no diffusion for the myelin compartment only (dark violet). For more information it is referred to Table 2 in the main manuscript. All values are presented as relative changes with respect to θ = 0°. The myelin compartment demonstrates the strongest orientation dependence for both R_1_ and R_2_ across all configurations, while extra-axonal and intra-axonal compartments show more modest angular variations.
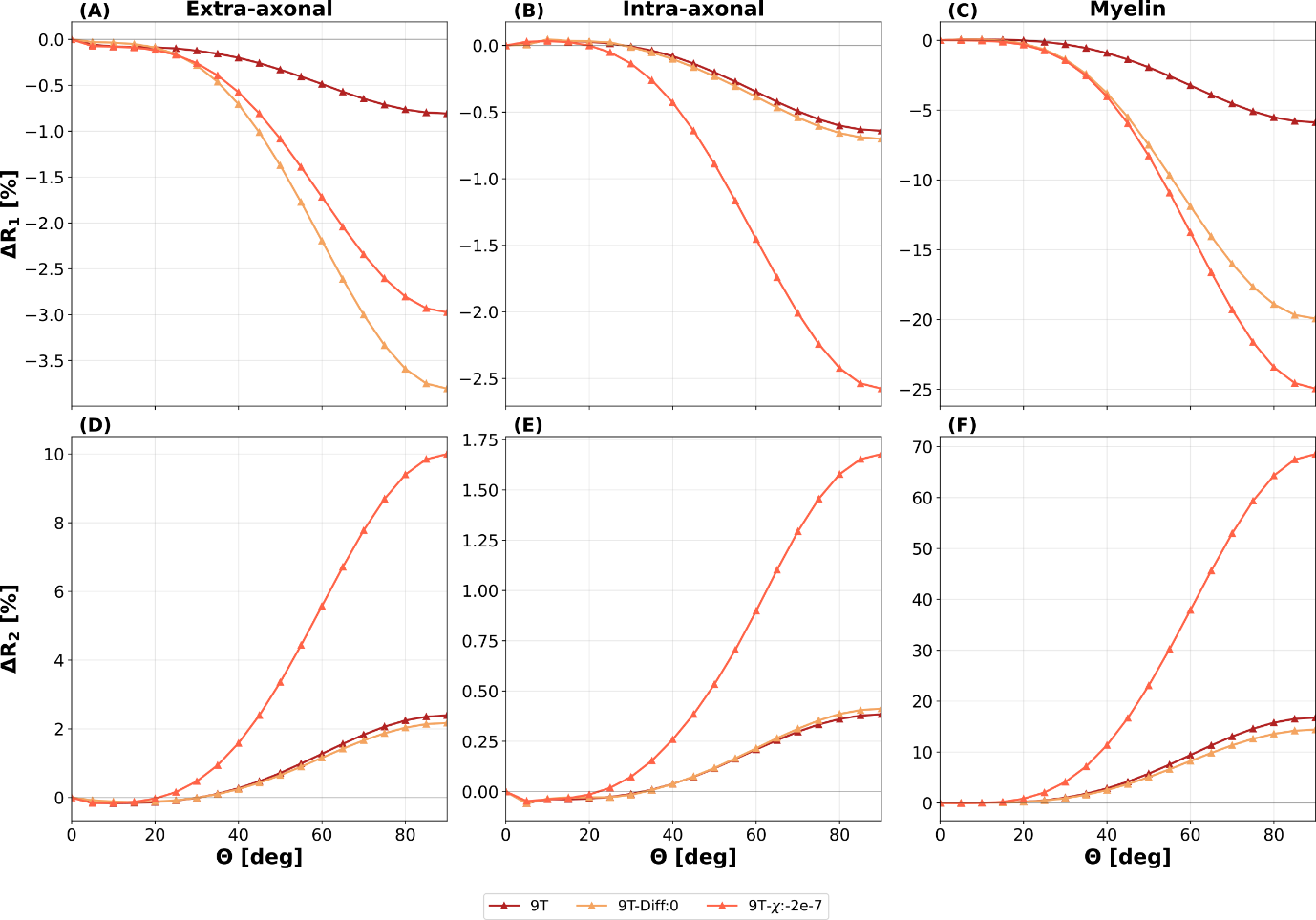
**Supporting Information Figure S5.** Compartmental analysis of SpinWalk simulations at 9.4 T. The relative orientation dependence of R_1_ (first row, **A-C**) and R_2_ (second row, **D-F**) is plotted as a function of fiber-to-field angle (θ) for the three individual compartments. Results are shown separately for extra-axonal (A, D), intra-axonal (B, E), and myelin (C, F) compartments. Different SpinWalk configurations are represented by distinct colors: default 9.4 T protocol (red), no diffusion for all compartments (light brown), and doubled magnetic susceptibility difference of myelin relative to intra-/extra-axonal water (orange). For more information it is referred to Table 2 in the main manuscript. All values are presented as relative changes with respect to θ = 0°. The orientation dependence is more pronounced for R_2_ compared to R_1_ for the extra-axonal and myelin compartments, latter demonstrating the strongest angular variations for both parameters.


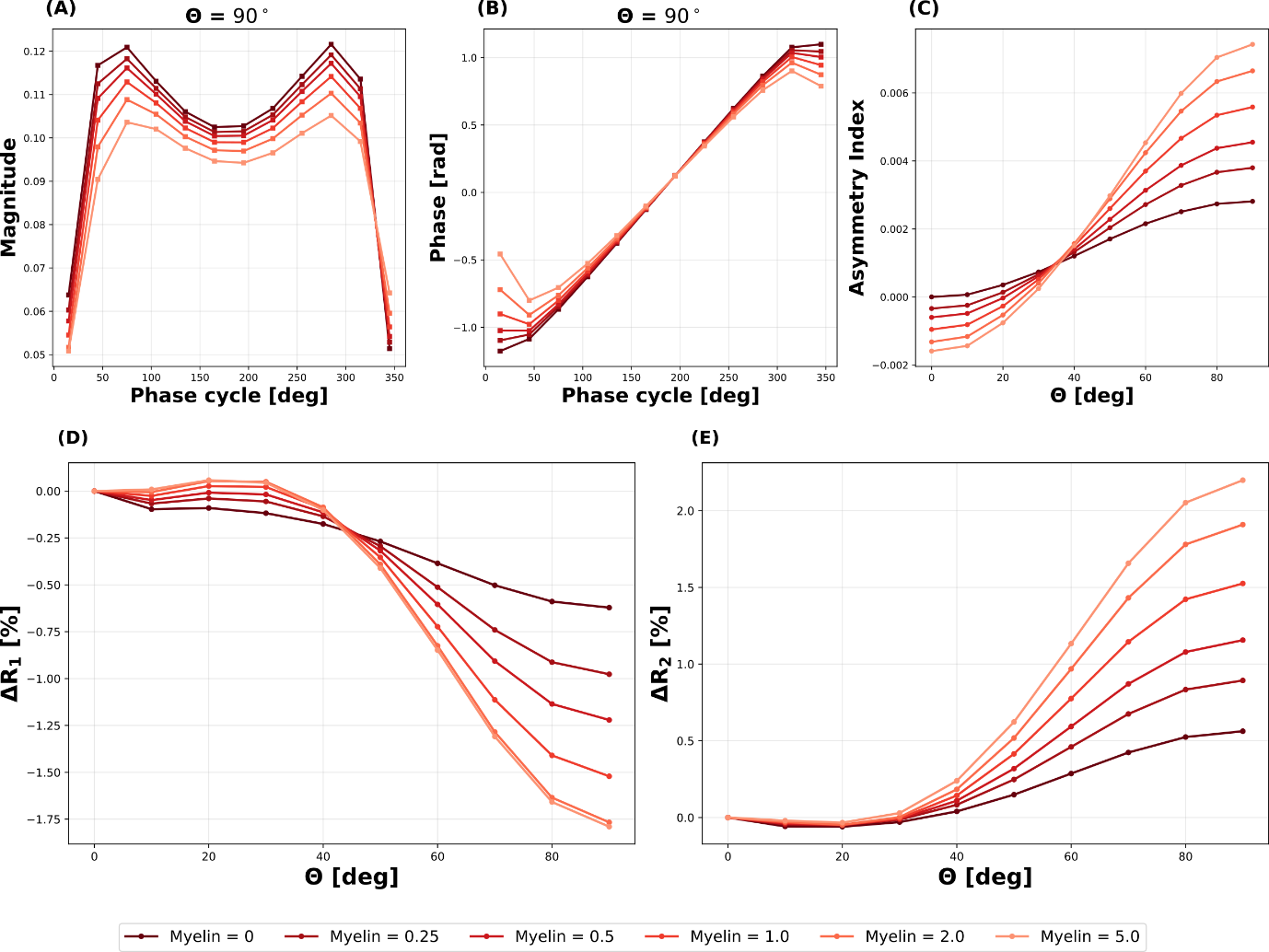
**Supporting Information Figure S6.** Effect of myelin proton density weighting relative to extra- and intra-axonal compartments on pc-bSSFP characteristics and relaxation anisotropy for SpinWalk simulations at 3 T. Magnitude (**A**) and phase (**B**) of the frequency response profiles at θ = 90° for different myelin proton density weightings, showing systematic changes in magnitude and phase characteristics. (**C-E**) Orientation dependence of asymmetry index (C), R_1_ (D), and R_2_ (E) across fiber-to-field angles (θ) for varying myelin proton density contributions. Different colors represent different myelin water fractions resulting from the respective proton density weightings, with lighter colors indicating increased weighting.


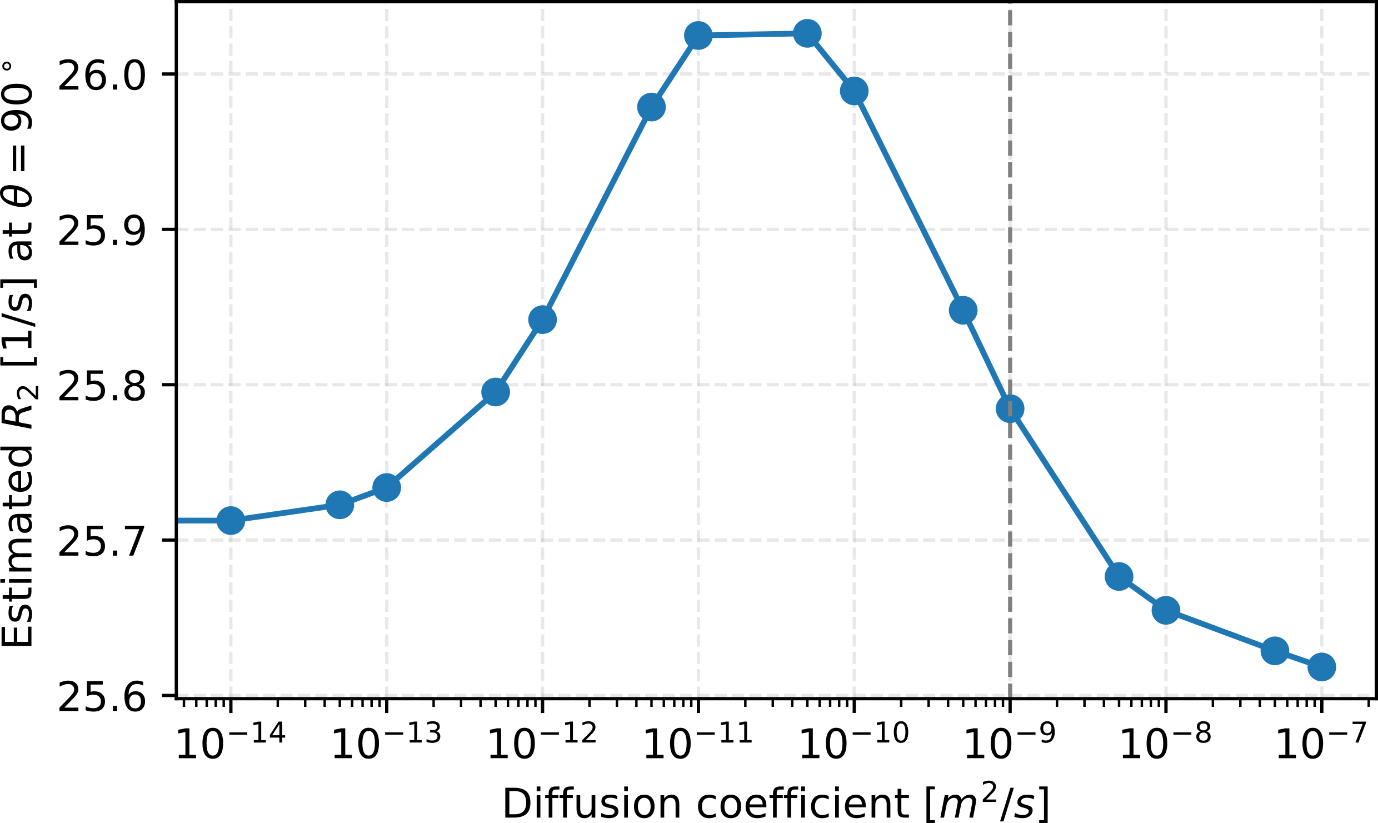
**Supporting Information Figure S7.** Impact of diffusion coefficient on estimated R_2_ at perpendicular fiber orientation (θ = 90°). SpinWalk simulations at 3 T with varying diffusion coefficients from 10^-14^ to 10^-7^ m²/s for intra- and extra-axonal compartments, with myelin diffusivity set one order of magnitude lower. The gray dashed vertical line indicates the diffusion coefficient (10^-9^ m²/s for extra- and intra-axonal, and 10^-10^ m²/s for myelin) used for the default simulation parameters in the main manuscript. R_2_ values increase with diffusion coefficients between 10^-14^ and 10^-11^ m²/s, plateau at intermediate values (10^-11^ to 10^-10^ m²/s), and decrease at higher diffusion coefficients (>10^-10^ m²/s), demonstrating the transition from the static dephasing to the motional narrowing regime.


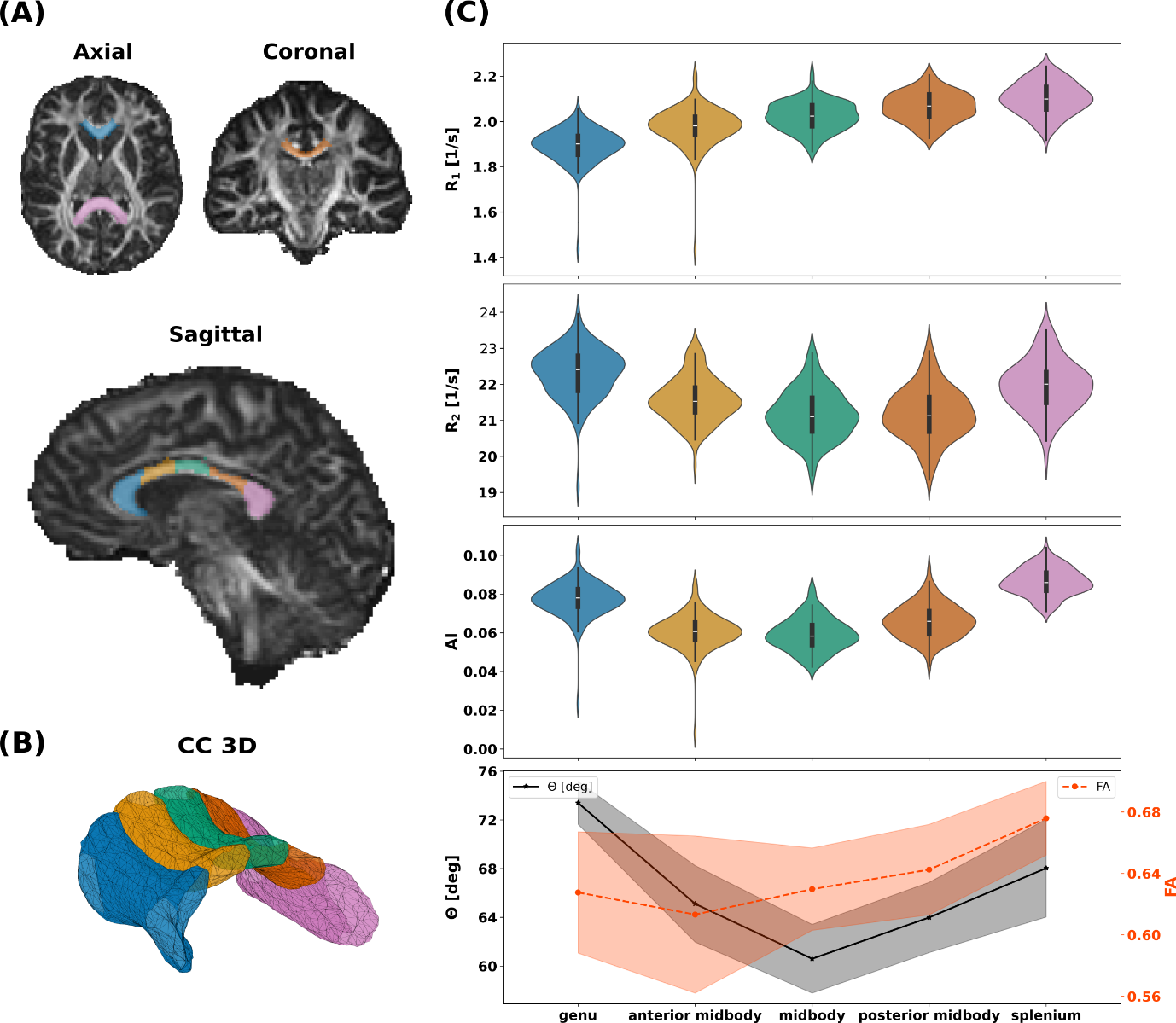
**Supporting Information Figure S8.** Corpus callosum (CC) analysis in five different segments. The CC was segmented into five equidistant regions along the anterior-posterior axis to evaluate R_1_, R_2_, and AI anisotropy at 3 T in a single WM structure. (**A**) Representative axial, coronal, and sagittal views of the segmented CC overlaid on anatomical T_1_-weighted data, demonstrating the anatomical delineation of the five segments. (**B**) 3D rendering of all five CC segments from the same subject as in (A), illustrating the anatomical curvature. (**C**) Violin and line plots, displaying the distribution of the assessed quantitative parameters (R_1_, R_2_, AI, θ, FA) in each segment, averaged across all subjects of the large-cohort 3 T study (n=107), progressing from the most anterior region (blue, genu) to the most posterior region (purple, splenium). The systematic variation in fiber-to-field angle (θ) across segments reflects the anatomical curvature of the CC relative to B_0_.

**REFERENCES**

1. Billot B, Greve DN, Puonti O, et al. SynthSeg: Segmentation of brain MRI scans of any contrast and resolution without retraining. *Medical Image Analysis*. 2023;86:102789. doi:10.1016/j.media.2023.102789

2. Wharton S, Bowtell R. Fiber orientation-dependent white matter contrast in gradient echo MRI. *Proceedings of the National Academy of Sciences*. 2012;109(45):18559-18564. doi:10.1073/pnas.1211075109

3. Hédouin R, Metere R, Chan KS, et al. Decoding the microstructural properties of white matter using realistic models. *NeuroImage*. 2021;237:118138. doi:10.1016/j.neuroimage.2021.118138

4. Xu T. Biophysical modeling of white matter in magnetic resonance imaging. 2017.

5. Andrews TJ, Osborne MT, Does MD. Diffusion of myelin water. *Magnetic Resonance in Med*. 2006;56(2):381-385. doi:10.1002/mrm.20945

6. Harkins KD, Dula AN, Does MD. Effect of intercompartmental water exchange on the apparent myelin water fraction in multiexponential *T*_2_ measurements of rat spinal cord. *Magnetic Resonance in Med*. 2012;67(3):793-800. doi:10.1002/mrm.23053

7. Endt S, Engel M, Naldi E, et al. In Vivo Myelin Water Quantification Using Diffusion–Relaxation Correlation MRI: A Comparison of 1D and 2D Methods. *Appl Magn Reson*. 2023;54(11-12):1571-1588. doi:10.1007/s00723-023-01584-1

8. Kulikova S, Hertz-Pannier L, Dehaene-Lambertz G, Poupon C, Dubois J. A New Strategy for Fast MRI-Based Quantification of the Myelin Water Fraction: Application to Brain Imaging in Infants. Schöpf V, ed. *PLoS ONE*. 2016;11(10):e0163143. doi:10.1371/journal.pone.0163143

9. Labadie C, Lee J, Rooney WD, et al. Myelin water mapping by spatially regularized longitudinal relaxographic imaging at high magnetic fields. *Magnetic Resonance in Med*. 2014;71(1):375-387. doi:10.1002/mrm.24670

10. Whittall KP, Mackay AL, Graeb DA, Nugent RA, Li DKB, Paty DW. *In vivo* measurement of *T*_2_ distributions and water contents in normal human brain. *Magnetic Resonance in Med*. 1997;37(1):34-43. doi:10.1002/mrm.1910370107

11. Xu T, Foxley S, Kleinnijenhuis M, Chen WC, Miller KL. The effect of realistic geometries on the susceptibility‐weighted MR signal in white matter. *Magnetic Resonance in Med*. 2018;79(1):489-500. doi:10.1002/mrm.26689

12. Wiggermann V, MacKay AL, Rauscher A, Helms G. In vivo investigation of the multi‐exponential *T*_2_ decay in human white matter at 7 T: Implications for myelin water imaging at UHF. *NMR in Biomedicine*. 2021;34(2):e4429. doi:10.1002/nbm.4429

13. Birkl C, Doucette J, Fan M, Hernández‐Torres E, Rauscher A. Myelin water imaging depends on white matter fiber orientation in the human brain. *Magnetic Resonance in Med*. 2021;85(4):2221-2231. doi:10.1002/mrm.28543

14. Hall MG, Clark CA. Diffusion in hierarchical systems: A simulation study in models of healthy and diseased muscle tissue. *Magnetic Resonance in Med*. 2017;78(3):1187-1198. doi:10.1002/mrm.26469

15. Oros-Peusquens AM, Loução R, Abbas Z, Gras V, Zimmermann M, Shah NJ. A Single-Scan, Rapid Whole-Brain Protocol for Quantitative Water Content Mapping With Neurobiological Implications. *Front Neurol*. 2019;10:1333. doi:10.3389/fneur.2019.01333

16. LoPachin R, Stys P. Elemental composition and water content of rat optic nerve myelinated axons and glial cells: effects of in vitro anoxia and reoxygenation. *J Neurosci*. 1995;15(10):6735-6746. doi:10.1523/JNEUROSCI.15-10-06735.1995

17. Mori S, van Zijl PCM, Oishi K, Faria AV. *MRI Atlas of Human White Matter*. 2nd ed. Burlington: Elsevier Science; 2010.

18. Yushkevich PA, Piven J, Hazlett HC, et al. User-guided 3D active contour segmentation of anatomical structures: Significantly improved efficiency and reliability. *NeuroImage*. 2006;31(3):1116-1128. doi:10.1016/j.neuroimage.2006.01.015
